# Cordycepin (3′-Deoxyadenosine) Suppresses Heat Shock Protein 90 Function and Targets Tumor Growth in an Adenosine Deaminase-Dependent Manner

**DOI:** 10.3390/cancers14133122

**Published:** 2022-06-25

**Authors:** Su-Chan Lee, Lujain Alaali, HyukJean Kwon, Mohammed Rigi, Charles G. Eberhart

**Affiliations:** Department of Pathology, Johns Hopkins University, Baltimore, MD 21205, USA; lalaali1@jhu.edu (L.A.); hkwon26@jhu.edu (H.K.); mrigi1@jhu.edu (M.R.)

**Keywords:** cordycepin, adenosine deaminase, uveal melanoma

## Abstract

**Simple Summary:**

Our study contributes to understanding the therapeutic effects of cordycepin in cancer. First, we find that the anticancer effects of cordycepin alone are only seen in tumor cell lines with low ADA expression or activity. This was true in multiple tumor types and may be applied as a predictive biomarker for the future treatment of patients. Second, we show that inhibition of ADA enhances cordycepin’s anticancer effects by blocking its conversion to 3′-deoxyinosine in uveal melanoma and several other tumors. This raises the possibility of clinical combination treatments with cordycepin and ADA inhibitors, and our data suggest that much lower doses of cordycepin are possible in this context. Third, we identified a new mechanism of action for cordycepin: inhibiting the function of a protein known as Hsp90.

**Abstract:**

Alterations in metabolism and energy production are increasingly being recognized as important drivers of neoplasia, raising the possibility that metabolic analogs could disrupt oncogenic pathways. 3′-deoxyadenosine, also known as cordycepin, is an adenosine analog that inhibits the growth of several types of cancer. However, the effects of cordycepin have only been examined in a limited number of tumor types, and its mechanism of action is poorly understood. We found that cordycepin slows the growth and promotes apoptosis in uveal melanoma, as well as a range of other hard-to-treat malignancies, including retinoblastoma, atypical teratoid rhabdoid tumors, and diffuse midline gliomas. Interestingly, these effects were dependent on low adenosine deaminase (ADA) expression or activity. Inhibition of ADA using either siRNA or pharmacologic approaches sensitized tumors with higher ADA to cordycepin in vitro and in vivo, with increased apoptosis, reduced clonogenic capacity, and slower migration of neoplastic cells. Our studies suggest that ADA is both a biomarker predicting response to cordycepin and a target for combination therapy. We also describe a novel mechanism of action for cordycepin: competition with adenosine triphosphate (ATP) in binding to Hsp90, resulting in impaired processing of oncogenic Hsp90 client proteins.

## 1. Introduction

3′-deoxyadenosine, also known as cordycepin due to its initial extraction from the fungus *Cordyceps militaris*, is an analog of adenosine that has been implicated in a range of biological processes [1]. Cordycepin is a medicinal component of several *Cordyceps* species, including *Cordyceps militaris* and *Cordyceps sinensis*, and as much as 0.84% cordycepin is found in ethanol extracts of *Cordyceps militaris* [2]. The bioavailability of cordycepin is 37 ± 11% after oral administration in rats [3]. A number of studies have demonstrated therapeutic effects in some tumor types. The mechanism(s) by which cordycepin inhibits tumor growth is only partially understood. In several studies, anti-oxidant activity and activation of the immune system were reported [4,5,6]. Cordycepin can bind to adenosine receptors and death receptors in glioma, melanoma, bladder, and non-small cell lung cancer cells, thereby promoting apoptosis [7,8,9,10]. It also binds to Epidermal Growth Factor Receptor (EGFR) and inhibits its downstream signaling pathway in human lung cancer cells [11]. However, these previous studies often investigated anticancer effects in only a few cancer cell lines. We therefore assessed the anticancer effects of 3′-deoxyadenosine in a large number of uveal melanoma cell lines, as well as other aggressive tumor types in which it has not yet been examined. 

Uveal melanoma, which arises from melanocytes located in the uveal tract of the eye, is the most common primary intraocular malignancy in adults. Mutations in the GNAQ and GNA11 genes, which encode guanine nucleotide-binding protein G(q) subunit alpha and guanine nucleotide-binding protein subunit alpha-11, are detected in more than 80% of primary uveal melanomas [12,13]. These mutations activate the rat sarcoma virus (RAS) pathway constitutively, increasing cancer cell proliferation, tumor progression, and growth [14]. Up to 40% of uveal melanomas metastasize hematogenously, primarily to the liver, and BRACA1-associated protein 1 (BAP1) mutations cause loss of function, as well as type 2 gene expression profiles, are associated with uveal melanoma dissemination and death [15,16,17]. Survival rates for patients with metastatic disease are extremely low, with the majority dying, and improved therapeutic strategies are clearly needed [18]. 

Adenosine deaminase (ADA), an essential enzyme for the purine salvage pathways, is expressed in a range of cell types [19]. ADA recognizes adenosine and 2′-deoxyadenosine and converts them to inosine and 2′-deoxyinosine, preventing their accumulation and inhibition of ribonucleotide reductase, which affects deoxyribonucleic acid (DNA) synthesis and repair [20]. Prior studies in infectious organisms, and a very limited number of tumor types, suggested that ADA also recognizes 3′-deoxyadenosine/cordycepin and converts it to 3′-deoxy-inosine, inhibiting its therapeutic effects [21,22,23]. 

In this study, we found that ADA expression and/or activity varies in uveal melanoma and other types of cancer cells and that higher levels are associated with less potent therapeutic effects of cordycepin. Both genetic and small molecule ADA inhibition sensitized cancer cells to cordycepin. We also report a novel mechanism of action for cordycepin involving engagement of the adenosine triphosphate (ATP) binding pocket of Heat shock protein 90 (Hsp90), thereby disrupting its function and leading to the degradation of client proteins such as hypoxia-inducible factor α (HIF-1α), protein kinase B (Akt), extracellular signal-regulated kinase (ERK), and EGFR.

## 2. Materials and Methods

### 2.1. Cell Culture, Plasmids and Reagents

Human uveal melanoma (92.1, Omm1, Mel202, Omm2.3, Omm2.5, MP46, and MM28) cell lines were kindly provided by Dr. J. Niederkorn (UT Southwestern Medical Center, Dallas, TX, USA) or purchased from the America Type culture Collection (Manassas, VA, USA). Rretinoblastoma (Y79, WERI-Rb1, and Rb143), atypical teratoid/rhabdoid tumor (AT/RT) (BT37 and BT12), diffuse midline gliomas H3 K27-altered, previously known as diffuse intrinsic pontine gliomas or “DIPG” (DIPG007, SF7761, and JHHDIPG1), pancreatic (PANC1), and breast cancer (MCF7) cell lines were generated in our laboratory [24] or purchased from the America Type culture Collection (Manassas, VA, USA). Human uveal melanoma and retinoblastoma cell lines were cultured in RPMI medium with 10% fetal bovine serum (FBS), penicillin/streptomycin (P/S), and L-glutamine previously described [25,26]. AT/RT cells were maintained in DMEM supplement with 10% FBS, P/S, and L-glutamine. DIPG cell lines were placed into cell culture in “EF media”: 30% Ham’s F12 media, 70% DMEM, 5% B27 regent, 1% L-glutamine, 1% antibiotic-antimycotic (Life Technologies, Frederick, MD, USA), 5 μg/mL heparin (Sigma-Aldrich, St. Louis, MO, USA), 20 ng/mL FGF, and 20 ng/mL EGF (Peprotech, Rocky Hill, NJ, USA) as previously described [24]. mCherry-Akt1 plasmid was provided by the Addgene. siRNA of ADA, b-actin, and hsp90 antibodies was purchased from Santa Cruz Biotechnology (Dallas, TX, USA). Akt, Erk, cleaved PARP, EGFR, and Hsp70 antibodies were provided by Cell Signaling Technology (Danvers, MA, USA). pET-15b-HIS-Hsp90 (FL), pET-15b-HIS-N- and C-terminal Hsp90 vectors were designed and provided from Genescript (Piscataway, NJ, USA). Cordycepin (3′-deoxyadenosine) was purchased from Sigma Aldrich (St. Louis, MO, USA) and Selleckchem (Houston, TX, USA).

### 2.2. Cell Growth Assays

Cells were seeded onto 96 cell plates at a density of 1000 cells per well and then incubated for 24 h. Cells were treated with vehicle or the indicated concentrations of cordycepin diluted in complete media for 5 days. Cells were further incubated with MTT solution (4 h incubation at 37 °C) or Cell-Titer blue solution (overnight incubation at 37 °C). The formazan products formed by addition of the MTT solutions were dissolved in dimethyl sulfoxide (DMSO), and the absorbance was measured at 570 nm. The fluorescence was measured at 560/600 nm for the Cell-Titer blue solution added plates. The data are presented as a percentage of the control group.

### 2.3. ADA Immunohistochemical Analysis and Activity Assays

The Adenosine Deaminase Activity Assay Kit (Sigma, St. Louis, MO, USA) used to identify activities of ADA. We followed a manufacturer’s guideline. Briefly, after incubation of fresh uveal melanoma cells with ADA assay buffer at 4 °C for 15 min. After centrifugation at 13,000 rpm, 4 °C for 10 min, supernatants were transferred to 96 cell culture dishes and incubated with ADA reaction mixture at 37 °C for 5 min, and then measured absorbance (O.D. 293 nm) in kinetic mode every thirty minutes at 37 °C. Immunohistochemical staining was performed on a tissue microarray containing four cores 0.6 mm in diameter from 65 human uveal melanomas using standard techniques and the following antibodies (ADA antibody 1:100, SC-28346, Santa Cruz Biotechnology, Dallas, TX, USA; biotinylated horse anti-mouse IgG, pk-6102 Vector lab, Burlingame, CA, USA). After microscopic analysis, ADA expression was scored by an ophthalmic pathologist (C.G.E.) in each tumor as negative, low, moderate, or high. 

### 2.4. Synergy Experiments

Values were obtained using MTT solutions after incubation of cells for 5 days. Data were analyzed using the ZIP method for drug combinations using the SynergyFinder web application online (https://synergyfinder.fimm.fi/; accessed on 20 August 2021) [27].

### 2.5. RNA Extraction and Quantitative Real-Time PCR

RNA extraction from cell lines was carried out using TRIzol (Invitrogen, Waltham, MA, USA). Quantitative real-time PCR (qPCR) was performed as previously described [28], with primer sequences were VEGF: forward 5′-TGCAGATTATGCGGATCAAACC-3′, reverse 5′-TGCATTCACATTTGTTGTGCTGTAG-3′; Actin forward: 5′-CCCAGCACAATGAAGATCAA-3′, reverse 5′-CGATCCACACGGAGTACTTG-3′. All reactions carried out in triplicate on QuantStudio3 (Applied Biosystems, Waltham, ME, USA), using SYBR Green (Applied Biosystems) fluorescent dye. The relative fold changing was calculated based on the formula R = 2 ^− (ΔCt sample − ΔCt control)^.

### 2.6. Soft Agar Assays (Anchorage-Independent Colony Formation Assays)

A total of 5000 sells per each well were mixed with agar solutions and were placed on base agar in 24-well plates. After solidification of the top agar, compounds diluted in complete medium were added to the agar, and it was incubated for 2–3 weeks until colonies were visualized in the vehicle group. The colonies were stained with MTT solution and imaged and counted by using the Image J program (NIH, Bethesda, MD, USA).

### 2.7. Anchorage-Dependent Colony Formation Assays

Cells were seeded onto 6-well plate at a density of 500 cells per well and then treated with different concentrations of cordycepin and ADA inhibitors for 2–3 weeks until colonies were visualized. Colonies were fixed with 100% methanol and stained with 0.002% crystal violet solutions (Sigma, St. Louis, MO, USA), and washed with deionized water two times. The colonies were imaged and counted using the ImageJ program (National Institutes of Health, Bethesda, MD, USA).

### 2.8. Migration Assays

Cells were seeded onto Transwells coated with gelatin only, and conditioned media from NIH3T3 fibroblast cells were placed in bottom wells. After incubation with vehicle, cordycepin, ADA inhibitors (EHNA and pentostatin), and combination treatment with cordycepin and an ADA inhibitor for 16 h, the migrated cells on the bottom of the gelatin-coated membrane were fixed with methanol and stained with crystal violet solutions. 

### 2.9. Western Blot Analysis

Cells were lysed in with RIPA buffer (Sigma, St. Louis, MO, USA) with protease inhibitor cocktails and phosphatase inhibitor cocktails (Roche, Indianapolis, IN, USA). Equal amounts of proteins were subjected to SDS-PAGE and electrically transferred to nitrocellulose or polyvinylidene difluoride (PVDF) membranes (Bio-Rad, Hercules, CA, USA). Membranes were blocked with 3% BSA in tris-buffered saline (TBS) containing 0.01% of Tween-20 for 1 h. The membranes were incubated with primary antibodies diluted in 3% BSA in TBST overnight at 4 °C with the following antibodies: ADA, Akt, Erk, EGFR, cleaved poly (ADP-ribose) polymerase (PARP), Hsp90, Heat shock protein 70 (Hsp70), and HIF-1α and then incubated secondary antibodies for 1 h at room temperature. Membranes were washed three times with TBST and visualized by using enhanced chemiluminescence (ECL). Original Western Blot figures shown in File S1. 

### 2.10. Annexin V Assay

Cells were seeded onto 12 well plates and then incubated for 1 day. Cells were treated with cordycepin and EHNA, and incubated for 2 days. The Muse cell analyzer and Muse annexin V and dead cell kit (Austin, TX, USA) were used to detect apoptotic cells. 

### 2.11. Animal Experiments

All animal experiments were performed according to protocols approved by the Johns Hopkins University Institutional Animal Care and Use Committee. Mice were fed standard mouse chow and water ad libitum and housed in temperature- and humidity-controlled facilities with a 12 h light/12 h dark cycle. For xenograft experiments, 92.1 and MP46 cells, which were diluted in equal amounts of medium and Matrigel, were injected subcutaneously into right and left flank of female nude mice. When tumor volume reached 50–100 mm^3^, the mice were randomly grouped and administered with vehicle (PBS), cordycepin (2–20 mg/kg), and pentostatin (1–2 mg/kg) every other day. Tumor growth was determined by measuring the short and long diameter of the tumor with a caliper, and body weight was also measured once a week to monitor toxicity. 

### 2.12. Molecular Docking

Calculations were performed based on the density functional theory (DFT) [29] at the B3LYP-D3 level of theory [30,31,32]. Optimization of cordycepin was carried out with the 6-31G** basis set [33] with several initial guess structures. Following geometry optimization, the electronic energies of the optimized cordycepin structure were recalculated with triple-ζ basis set cc-pVTZ (-f) [34]. Vibrational frequency was calculated at the same level of theory as the geometry optimization. Entropy correction along with the zeropoint vibrational energy was considered for proper thermodynamic approximations. Based on the gas phase scaffolds, solvation correction energies were deduced. Self-consistent reaction field (SCRF) [35,36,37] approximations were used to calculate the linearized Poisson–Boltzmann equations with the dielectric constant ε. The solvation energy used in the system was treated with water (ε = 78.4). The Gibbs free energies in solution phase were computed as the following equations:

G(sol) = G(gas) + G(solv)(1)

G(gas) = H(gas) − TS(gas)(2)

H(gas) = E(SCF) + ZPE(3)

### 2.13. Statistical Analysis

The data are presented as the means ± standard deviation (SD) or standard error of mean (SEM). All in vitro experiments were independently performed, and a representative result is presented. The data were calculated or analyzed with Graph Pad Prizm (San Diego, CA, USA). Statistical significance was determined using a one-way ANOVA test. A *p* value of less them 0.05 was considered significant. 

## 3. Results

### 3.1. Cordycepin Slows Growth of Uveal Melanoma Cells with Low Adenosine Deaminase

**Figure 1:**
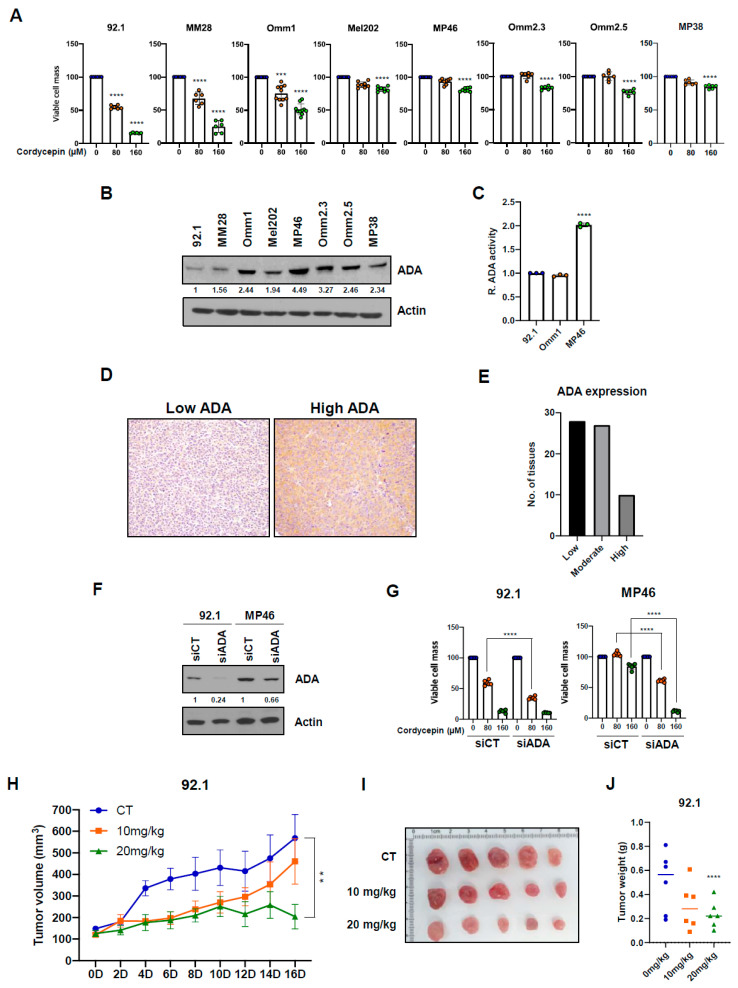
ADA decreases anticancer effects of cordycepin in uveal melanoma. (**A**) Growth of viable cell mass after cordycepin treatment. Uveal melanoma cells were treated with vehicle, 80, and 160 µM of cordycepin, and after 5 days incubation viable cell mass was measured using fluorescence. (**B**) Adenosine deaminase (ADA) protein expression levels varied uveal melanoma cells lines on western blots. (**C**) ADA enzymatic activity in uveal melanoma. (**D**,**E**) Immunohistochemical ADA protein expression in human uveal melanomas on tissue microarrays. (**F**,**G**), ADA protein expression (**F**) and cell growth (**G**) in 92.1 and MP46 uveal melanoma cells after siRNA-based knockdown. Values represent mean ± SD of experiments conducted in sextuplicate. **** *p* < 0.0001 by one-way ANOVA analysis of variance compared with control group. (**H**) Antitumor effects of cordycepin in mice bearing xenograft tumors of 92.1 uveal melanoma cells. (**I**) Pictures of xenografts from 92.1 uveal melanoma cells. (**J**) Tumor weight in vehicle, 10 mg/kg and 20 mg/kg cordycepin treatment group. Values represent mean ± SEM of experiments. CT, control; 10 mg/kg, cordycepin 10 mg/kg b.w. treatment; 20 mg/kg, cordycepin 20 mg/kg b.w. treatment. ** *p* < 0.01, *** *p* < 0.001, **** *p* < 0.0001 by one-way ANOVA analysis of variance compared with control group.

We assessed the potency of cordycepin against a panel of uveal melanoma cell lines and found significant inhibition of growth of viable cell mass over 5 days in 92.1, MM28, and Omm1 cells at both 80 µM (24–45% reduction) and 160 µM (48–84% reduction; Figure 1A, Supplementary Figure S1A). In contrast, in the remaining five lines, no significant growth reduction was seen with 80 µM cordycepin, while the higher concentration slowed growth by only (15–23%). Previous studies suggested that the effects of cordycepin could depend on ADA levels or activity, as this enzyme can catalyze the hydrolysis of cordycepin [38,39]. Therefore, we hypothesized that ADA expression or activity levels could modulate the anticancer effects of cordycepin in uveal melanoma cell lines. 

ADA protein was differentially expressed in our uveal melanoma cell lines (Figure 1B). Levels on western blot were lowest in the 92.1 and MM28 cell lines showing the greatest sensitivity to cordycepin. While Omm1 cells showed significantly decreased growth after treatment with cordycepin, ADA protein levels were relatively high. Therefore, we evaluated ADA enzymatic function in several of the lines, which revealed a low ADA activity level in Omm1 cells, similar to that in 92.1 cells, and lower than treatment-resistant MP46 cells (Figure 1C). Finally, we examined ADA protein in primary human uveal melanoma specimens by immunohistochemical analysis of a tissue array, which confirmed heterogeneous expression levels in primary tumors. We found that 28 uveal melanomas had a low expression, 27 had a moderate expression, and 10 had high expression (Figure 1D,E). These data suggest that ADA expression and activity can vary between uveal melanoma and that the effects of cordycepin may depend on this enzyme.

To further assess the importance of ADA in modulating the effects of cordycepin on tumor growth, we used siRNA targeting ADA on ADA-low (92.1) and ADA-high (MP46) lines. We achieved almost complete knockdown of ADA protein in the 92.1 cells and reduced levels in MP46 cells to near the baseline seen in 92.1 (Figure 1F). The further reduction of ADA in 92.1 significantly improved their response to 80 µM cordycepin, while partial ADA knockdown in the MP46 line resulted in significantly greater growth reductions over 5 days after both 80 and 160 µM cordycepin treatment (Figure 1G). 

Finally, we assessed the antitumor effect of cordycepin in uveal melanoma xenograft models established in immunocompromised (nude) mice. Consistent with our in vitro results in uveal melanoma with reduced ADA expression, treatment with 20 mg/kg body weight (b.w.) of cordycepin significantly suppressed the tumor growth in 92.1 uveal melanoma xenografts with low ADA protein (Figure 1H–J). In contrast, as discussed below, the growth of high ADA MP46 xenografts was less sensitive to 20 mg/kg b.w. of cordycepin treatment as compared to the control group. The mean weight of xenografts treated with 10 mg/kg cordycepin was not significantly different, while the mean weight of those treated with 20 mg/kg was 54% less than controls, significantly (Figure 1J). When taken together, these results indicate that ADA expression or activity could be a predictive biomarker for cordycepin treatment of uveal melanoma.

### 3.2. Cordycepin Decreases Tumor Migration and Colony Formation

**Figure 2:**
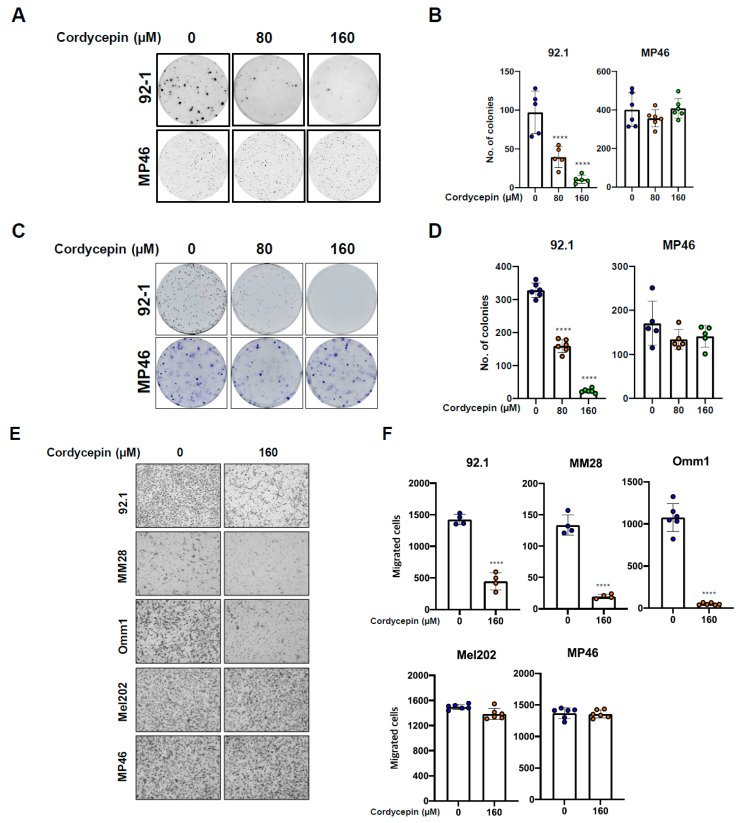
Cordycepin inhibits colony formation and migration abilities of uveal melanoma cells in an ADA dependent manner. (**A**,**B**) Effects of cordycepin treatment on soft agar colony formation of uveal melanoma cells. (**C**,**D**) the effect of cordycepin treatment on the anchorage-dependent colony formations. (**E**,**F**) Inhibition of migration abilities of uveal melanoma cells by treatment with 160 µM cordycepin. Values represent mean ± SD of experiments conducted in sextuplicate. **** *p* < 0.0001 by one-way ANOVA analysis of variance compared with control group.

We next assessed the effects of cordycepin on colony formation of uveal melanoma cells using a semi-solid soft agar. A total of 92.1 uveal melanoma cells with low ADA expression showed a significant, dose-dependent decrease in colony formation after 80 µM and 160 µM cordycepin treatment (Figure 2A,B). The 80 and 160 µM cordycepin treatment also significantly inhibited soft agar colony formation in Omm1 cells with low ADA enzymatic activity (Supplementary Figure S1B,C). However, in several high ADA uveal melanoma lines, Mel202, MP46, and MP38, there were no significant differences in soft agar colony formation at these levels of cordycepin (Figure 2A,B; Supplementary Figure S1B,C). We also performed anchorage-dependent colony formation assays with uveal melanoma cells. As was seen with cells grown in soft agar, 80 and 160 µM of cordycepin strongly decreased monolayer colony formation in low ADA (92.1 and MM28) uveal melanoma cells but not in MP46 and Mel202 high ADA cells (Figure 2C,D; Supplementary Figure S1D,E).

Next, we evaluated the effect of cordycepin on uveal melanoma migration in vitro, since previous research showed that it suppressed the migration of bladder cancer cells [40]. Treatment with 160 µM of cordycepin for 16 h significantly decreased migration of the 92.1, MM28, and Omm1 uveal melanoma cell lines. However, there were no effects of this concentration on Mel202 and MP46 (Figure 2E,F). These data suggest that cordycepin effectively inhibits the colony forming and migration abilities of uveal melanoma with low ADA expression or activity.

### 3.3. Targeting ADA Promotes Anticancer Effects of Cordycepin In Vitro and In Vivo

**Figure 3:**
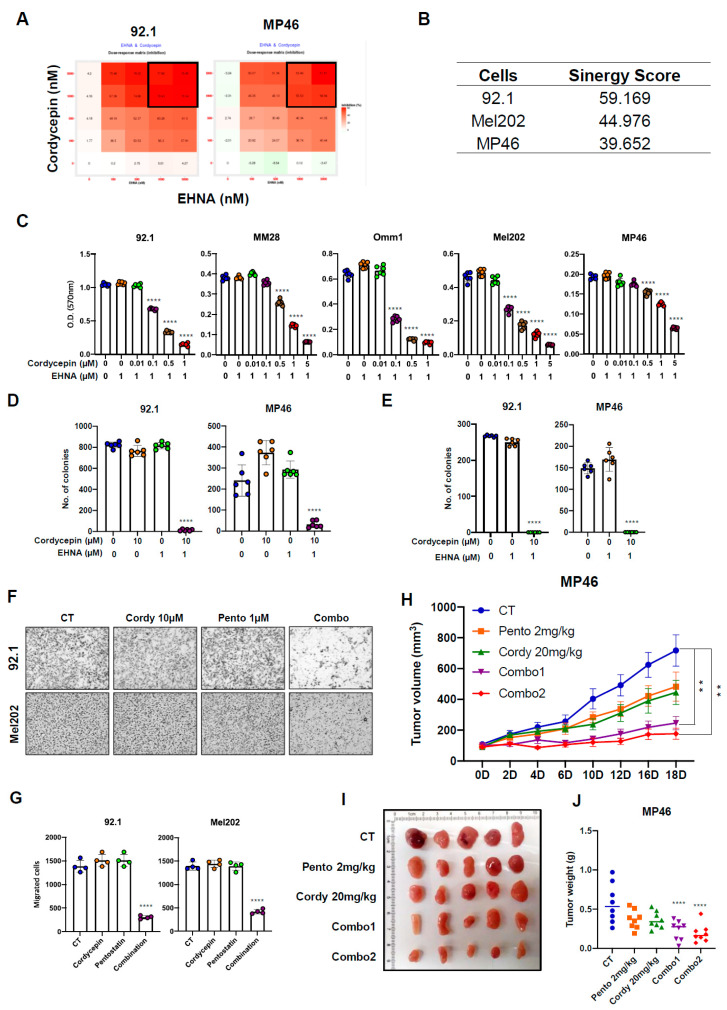
Targeting ADA synergistically enhances anticancer effects of cordycepin in uveal melanoma. (**A**,**B**) Synergistic effects (**A**) and scores (**B**) of cordycepin and EHNA in 92.1, Mel202 and MP46 cells. (**C**) Combination treatment of 3′-deoxyadenosine and EHNA in uveal melanoma cells for cell growth assays. (**D**,**E**) Effects of cordycepin and EHNA on anchorage independent (**D**) and dependent (**E**) colony formation abilities in uveal melanoma. (**F**,**G**) Inhibition of cell migrations of uveal melanoma by combination treatment of 1 µM pentostatin and 10 µM cordycepin. The cells were seeded onto the Transwells coated with gelatin. After incubation for 12 h, the migratory cells on the bottom of the membrane were stained with crystal violet solution and counted. Values represent mean ± SD of experiments conducted in quadruplicate, or sextuplicate or octuplicate. **** *p* < 0.0001 by one-way ANOVA analysis of variance compared with control group. (**H**) Antitumor effects of cordycepin in mice bearing xenograft tumors of MP46 uveal melanoma cells. (**I**) Pictures of xenografts from MP46 uveal melanoma cells. (**J**) Tumor weights in vehicle- or compound treated mice. Values represent mean ± SEM of experiments. CT, control; Pento 2 mg/kg, pentostatin 2 mg/kg b.w. treatment; 3′-DA 20 mg/kg; Combo 1, combination treatment with pentostatin 1 mg/kg b.w. and 3′-deoxyadenosine 2 mg/kg b.w.; Combo 2, combination treatment with pentostatin 2 mg/kg b.w. and cordycepin 2 mg/kg b.w. ** *p* < 0.01, **** *p* < 0.0001 by one-way ANOVA analysis of variance compared with control group.

To determine if combination therapy could further sensitize uveal melanoma cells to cordycepin, we used pharmacologic inhibition of ADA. EHNA (erythro-9-(2-hydroxy-3-nonyl) adenine) and pentostatin are adenosine deaminase inhibitors that are non-toxic to cells [41,42,43]. Thus, we examined whether EHNA and pentostatin treatment would enhance the anticancer effects of cordycepin on uveal melanoma. The SynergyFinder program was used to choose a concentration of cordycepin and ADA inhibitors in uveal melanoma cells (Figure 3A). This resulted in synergy scores of 59 (92.1), 45 (Mel202), and 40 (MP46), reflecting the average excess response due to drug interactions (Figure 3B). In three uveal melanoma cell lines, 1–5 μM cordycepin concentrations in the presence of 1–5 μM EHNA resulted in maximal growth inhibition (Figure 3A). As shown in Figure 3C and Supplemental Figure S2A, combination treatment with even very low levels of the drugs could significantly enhance growth inhibition of uveal melanoma line, including those with high levels of ADA, as compared to the monotherapy studies with much higher concentrations of cordycepin shown in Figure 1A. A combination of 1 µM cordycepin and 1 µM EHNA resulted in a 68% reduction of growth on average in the five lines tested, while 1 µM cordycepin and 1 µM pentostatin inhibited growth by 69% on average.

As was true for adherent growth, combination treatment with cordycepin and EHNA markedly inhibited anchorage-independent and -dependent colony formation in all uveal melanoma cell lines (Figure 3D,E; Supplementary Figure S2B–E). In addition, Combination treatment with 10 µM of cordycepin and 1 µM of an ADA inhibitor (EHNA or pentostatin) significantly inhibited migrated cells in all uveal melanoma cell lines (Figure 3F,G; Supplementary Figure S2F,G). These results suggest that even a low concentration of cordycepin effectively inhibits colony formation and migration abilities in uveal melanoma when ADA activity is suppressed pharmacologically. 

We next evaluated in vivo murine xenograft models. While 20 mg/kg body weight cordycepin alone did not suppress tumor growth significantly, combination treatment with a much lower dose of cordycepin (1 or 2 mg/kg) and 1 mg/kg of pentostatin markedly inhibited the tumor growth in the MP46 xenograft-bearing mice (Figure 3H–J). Previous studies showed that cordycepin treatment does not affect mice’s body weight, suggesting low toxicity [23]. During the treatment, we also did not observe any difference in body weight in mice administered cordycepin alone, or after combination treatment with cordycepin and pentostatin, as compared with the control group (Supplementary Figures S1F and S2H). When taken together, these results indicate that the inhibition of ADA function enhances antitumor effects of cordycepin in uveal melanoma with both low ADA and high ADA expressed.

### 3.4. Cordycepin Induces Apoptotic Cell Death in Uveal Melanoma

**Figure 4:**
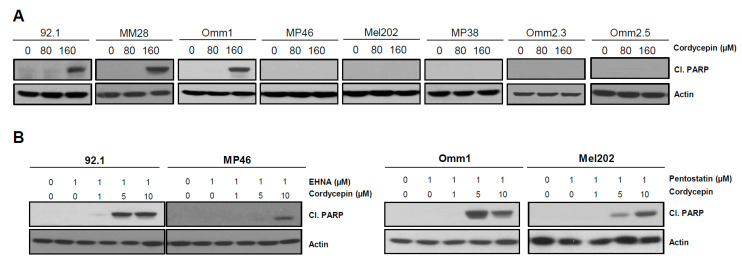

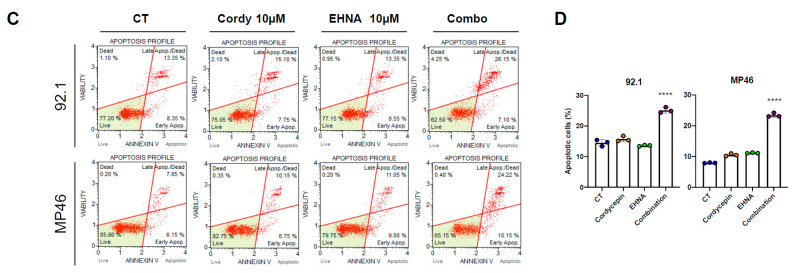
Apoptotic induction in uveal melanoma cells. (**A**,**B**) Uveal melanoma cells were treated with cordycepin only, or combination treatment with 3′-deoxyadenosine and ADA inhibitors, and the expression cleaved poly-(ACP-ribose) polymerase (PARP) was analyzed by Western blot analysis. (**C**,**D**) Cells were treated with 10 µM cordycepin and 1 µM EHNA for 48 h, and the distribution of Annexin V positive cells was analyzed by flow cytometry. Values represent mean ± SD of experiments conducted in triplicate. **** *p* < 0.0001 by one-way ANOVA analysis of variance compared with the control group.

To investigate whether cordycepin alone could induce apoptosis in uveal melanoma, we assessed cleaved Poly (ADP-ribose) polymerase (PARP) protein expression after 80 and 160 µM cordycepin treatment of uveal melanoma cells. Cordycepin single treatment increased cleaved PARP protein expression in ADA low (92.1, MM28, Omm1) uveal melanoma cells but not in five lines with high ADA protein expression (Figure 4A). However, the combination treatment with cordycepin (1–10 µM) and an ADA inhibitor (1 µM) markedly increased cleaved PARP protein in all lines tested, including those with higher ADA (Figure 4B). The effect of cordycepin on apoptotic activity was also examined using the annexin V flow cytometric assay. This confirmed that combination treatment with 10 µM cordycepin and 1 µM EHNA significantly increased apoptotic cells in uveal melanoma (Figure 4C,D). These results suggest that the antitumor effects of cordycepin in uveal melanoma are at least in part due to an increase in apoptosis.

### 3.5. Cordycepin Disrupts the Function of Hsp90 and Induces Degradation of Its Client Proteins

We next investigated the mechanism by which cordycepin targeted uveal melanoma. Previous studies have suggested cordycepin can directly bind proteins, thereby regulating the activation of several signaling pathways [7,11,44]. Because it is an analog of adenosine, we hypothesized that cordycepin might directly interact with the ATP binding pocket of Hsp90 and modulate its function. 

**Figure 5:**
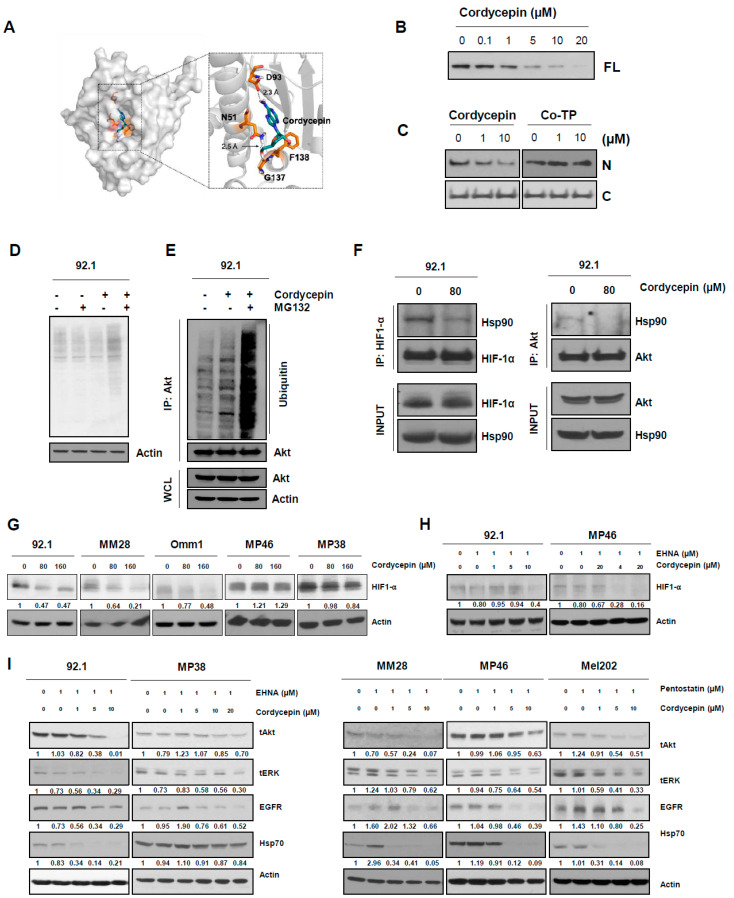
Cordycepin binds Hsp90 and disrupts its function, resulting in degradation of client proteins. (**A**) Visualization of possible interactions between the N-terminal of HSP90 (PDB: 6GPT) [52] and cordycepin within the HSP90 hydrophobic pocket (e.g., ATP binding site) observed by docking simulations. Non-polar hydrogens and amino acid residues apart from the key residues known for ATP binding (e.g., N51, D93, G137 and F138) [53,54] were omitted for clarity. (**B**,**C**) Competition assay with cordycepin and ATP (B and C left) and with cordycepin triphosphate and ATP ((**C**) right) using human recombinant full-length Hsp90 (FL), N-terminal and C-terminal domain. (**D**) 92.1 cells were treated with vehicle, 10 µM MG-132, and 80 µM cordycepin. (**E**) Co-immunoprecipitation assay using an anti-Akt antibody with vehicle, 80 µM cordycepin, and 80 µM cordycepin/10 µM of MG-132 (combination) then incubated for 6 h. (**F**) Cells were treated by 80 µM cordycepin only under hypoxic conditions (left) or 20% oxygen condition (right) for 1.5 h. Total cell lysates were prepared and Co-immunoprecipitated (Co-IP) with anti-HIF-1α or Akt antibodies. The interaction between client protein (HIF-1α or Akt) and Hsp90 was analyzed by Western blot analysis. (**G**,**H**) Cells were treated with cordycepin, or combination (4–20 µM cordycepin3′-deoxyadenosine and 1 µM EHNA) for 1.5 h under the hypoxic condition. HIF-1α protein level was analyzed by Western blot. (**I**) Cells were treated with cordycepin in the presence of 1 µM EHNA (**left**) or pentostatin (**right**) for 2 days. Total Akt, total ERK, EGFR, and Hsp70 protein expression levels were analyzed by Western blot.

A computational docking modeling was used to examine the possibility of cordycepin interacting with the ATP binding pocket of Hsp90. An ATP binding site is present in the N-terminal domain, and a plausible binding mode between cordycepin and the N-terminal of Hsp90 (PDB: 6GPT^1^) was observed using docking simulations (Figure 5A). We were able to identify two possible hydrogen bonds between the amino acid residues in the hydrophobic pocket of Hsp90 and our ligand: (i) a hydrogen bond formed between the sidechain of N51 where the primary amine acts as a proton donor and the 5′OH cordycepin which acts as a proton acceptor with a length of 2.51 Å; (ii) a carboxylate group within the sidechain of D93 interacting with the proton of the primary amine in cordycepin with a length of 2.28 Å. As cordycepin appears to dock stably in this hydrophobic pocket, our simulations suggest the possibility of competitive inhibition of Hsp90 by cordycepin over ATP.

Next, we experimentally assessed the capacity of cordycepin to binding Hsp90 protein. While the ATP binding pocket is located in the N-terminal domain of Hsp90, the C-terminal domain also has a nucleotide-binding region, and recombinant N- and C- terminal domains of Hsp90 were both used to evaluate the affinity of cordycepin. Full-length (FL) recombinant human Hsp90, or the N- and C-terminal domains, were preincubated with cordycepin and cordycepin triphosphate (Co-TP), respectively, and then ATP-linked agarose resins were added. ATP binding to the FL and N terminal domains of Hsp90 was clearly diminished by treatment of cordycepin in a dose-dependent manner (Figure 5B,C). However, no ATP binding competition was observed in the C-terminal domain (Figure 5C), suggesting cordycepin interactions may be limited to the N-terminal ATP binding pocket of Hsp90. 

Prior studies also suggested that cordycepin triphosphate, one of the metabolites of cordycepin, can regulate the NF-kB signaling pathway [45]. Therefore, we assessed whether cordycepin triphosphate would compete with ATP. However, cordycepin triphosphate treatment did not affect the ATP binding to the N-terminal domain of Hsp90 (Figure 5C, right). 

Hsp90 inhibition results in misfolded client proteins that are subsequently ubiquitinated and degraded by the proteasome. To confirm that the binding of cordycepin to Hsp90 was associated with impaired processing of client proteins, we evaluated global protein ubiquitination after treatment with cordycepin for 2 h. In 92.1 cells, 160 µM cordycepin treatment and combination treatment with 10 µM cordycepin and 1 µM EHNA increased ubiquitination in the presence of MG-132 (Figure 5D and Supplementary Figure S3A). Moreover, polyubiquitination of Akt, a client protein of Hsp90, was evident in cordycepin-treated 92.1 cells in which proteasome machinery was inactivated by MG-132 (Figure 5E). These findings suggest that cordycepin deregulates Hsp90 protein, resulting in induction of ubiquitin-mediated proteasomal degradation of client proteins such as Akt.

We next further examined specific client proteins of Hsp90, including HIF-1α and Akt. Since HIF-1α is induced under hypoxic conditions easily [46,47,48,49,50], first co-immunoprecipitation assays were performed to investigate interactions between Hsp90 and HIF-1α. Treatment with 80 µM cordycepin decreased protein interaction between Hsp90 and HIF-1α in 92.1 uveal melanoma cells (Figure 5F left), and combination treatment with 10 µM cordycepin and 1 µM EHNA also prominently reduced binding of these proteins (Supplemental Figure S3B). A total of 80 µM cordycepin treatment also inhibited protein interaction between Hsp90 and Akt (Figure 5E right). Consistent with the role of Hsp90 in promoting HIF-1α and Akt stability, 80 µM and 160 µM cordycepin treatment significantly decreased protein levels of HIF-1α, Akt, ERK, and EGFR in ADA-low (92.1, MM28, Omm1) uveal melanoma cell lines, but not in those with high ADA (MP46 and MP38; Figure 5E; Supplemental Figure S3B,C). 

These Hsp90 client proteins showed similar changes after treatment with cordycepin and an ADA inhibitor. As shown in Figure 5F,G, HIF-1α, Akt, ERK, and EGFR protein levels all decreased in uveal melanoma cell lines after combination treatment. Some previous studies have reported that Hsp90 inhibitors can induce compensatory expression of Hsp70 protein [51], but in our uveal melanoma lines, Hsp70 protein levels were reduced to varying degrees after treatment with cordycepin and EHNA or pentostatin (Figure 5G).

We evaluated VEGF mRNA, which is regulated by HIF-1α. A total of 160 µM cordycepin treatment decreased mRNA expression of VEGF in ADA-low uveal melanoma, while it was not decreased in high ADA uveal melanoma cells (Supplementary Figure S4A). Next, the anti-angiogenic effects of cordycepin treatments in vivo using these xenografts were investigated. We noted that the 92.1 xenografts treated with 20 mg/kg cordycepin were paler than those in the control group (Figure 1I and Figure 3I), suggesting that vascularity might be lower. This would be consistent with the changes to HIF-1α and VEGF documented in our in vitro studies. Indeed, western analysis of protein extracted from the xenografts confirmed lower levels of the vascular endothelial marker VEGF, supporting the possibility of anti-angiogenic effects of this therapy (Supplementary Figure S4A,B).

When taken together, these results indicate that cordycepin directly binds to Hsp90 and blocks its function, resulting in inhibition of the interaction between Hsp90 and multiple client proteins.

### 3.6. ADA-Dependent Effects of Cordycepin in Other Cancers

**Figure 6:**
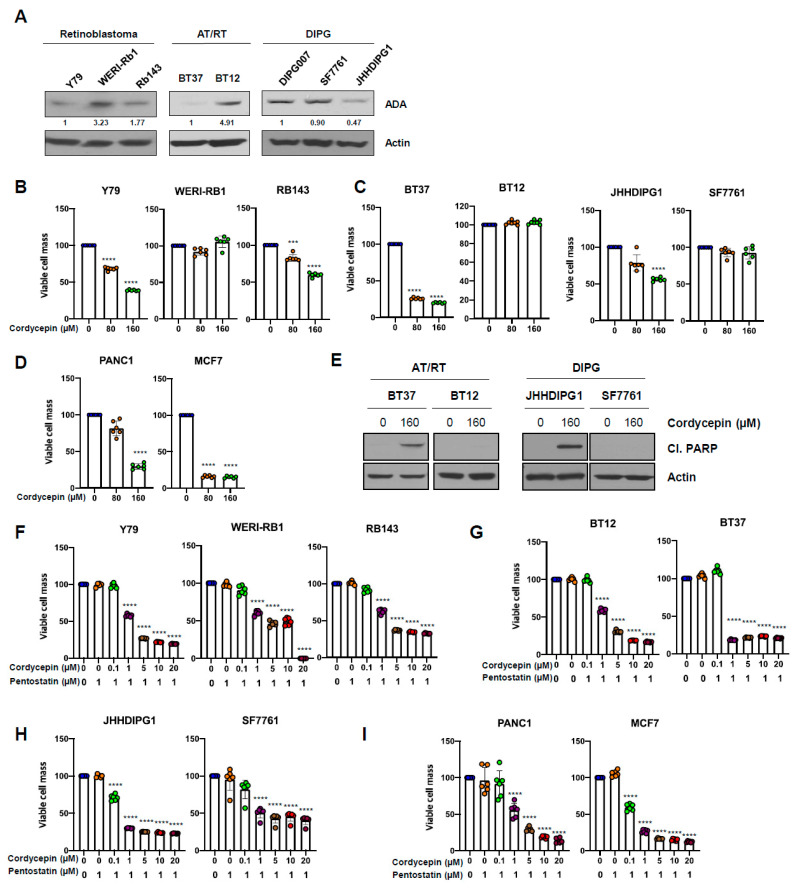
Anticancer effects of cordycepin in other aggressive cancer cells. (**A**) ADA protein expression levels were measured by Western blot in retinoblastoma, AT/RT, and DIPG cell lines. (**B**–**D**) Cells were treated with for 5 days and then cell titer blue was used to measure viable cells. (**E**) Cells were treated with cordycepin for 2 days, and then cleaved PARP protein level was evaluated by Western blot. (**F**–**I**) Cells were treated with cordycepin and 1 µM of pentostatin for 5 days and then cell titer blue was used to measure viable cells. Values represent mean ± SD of experiments conducted in sextuplicate. *** *p* < 0.001, **** *p* < 0.0001 by one-way ANOVA analysis of variance compared with control group.

We next assessed whether the anti-cancer effects of cordycepin on other types of cancer cells would be similar to those in uveal melanoma. Western blot analysis of ADA protein in retinoblastoma (Y79, WERI-Rb1, and Rb143), malignant atypical teratoid/rhabdoid tumors of the brain (AT/RT; BT37 and BT12), and diffuse midline gliomas, H3K27M mutant of the brain (DIPG007, SF7761, and JHHDIPG1) lines showed variable levels similar to those in uveal melanoma (Figure 6A). Treatment with cordycepin (80 and 160 µM) significantly suppressed the growth of low ADA cell lines, including Y79, RB143, BT37, JHHDIPG1, PANC1, and MCF7, while WERI-Rb1, BT12, and SF7761 lines with high ADA were resistant to cordycepin treatment at these levels (Figure 6B–D). Treatment with 160 µM cordycepin also increased cleaved PARP protein in low ADA active AT/RT (BT37) and diffuse midline glioma H3 K27-altered cells (JHH-DIPG1), consistent with an increase in apoptosis (Figure 6E). However, we did not detect cleaved PARP in high ADA AT/RT (BT12) and diffuse midline glioma cells (SS7761; Figure 6E). Combination treatment with cordycepin (1–20 µM) and 1 µM of pentostatin also induced significantly decreased cell growth in cancer cell lines with both high and low ADA levels (Figure 6F–I). These data indicate that the treatment of cordycepin not only affects the anti-cancer activities of uveal melanoma but also the anti-cancer activities of various different types of cancers depending on ADA activity.

## 4. Discussion

In this study, we report that in a range of malignancies, including uveal melanoma and several types of aggressive brain tumors, high ADA expression or activity in cell line models is associated with a dramatically reduced therapeutic response to cordycepin. 

Our work builds on previous reports, which found anticancer efficacy of cordycepin in murine glioma, cutaneous melanoma, and lung carcinoma cells, as well as human bladder and lung cancer cells, although most of these studies evaluated only one or two cell lines [7,8,9,10,11]. These earlier studies suggested an IC_50_ for cordycepin of approximately 160 µM; however, in our initial uveal melanoma experiments with 80 and 160 µM doses, we found great variation in efficacy with respect to a decrease in cell growth. Previous work had shown that 3′-deoxyadenosine/cordycepin is converted to 3′-deoxyinosine by the ADA enzyme [3,19,20], and we hypothesized that different expression levels or activity of ADA might explain the heterogeneous response in uveal melanoma cells. Very recently, it has been shown that a new analog of cordycepin, NUC-7738, which is resistant to ADA, has anti-cancer activities in cancer cells, providing additional evidence for the importance of ADA in modulating the effects of this adenosine analog [45]. 

Indeed, in uveal melanoma cell lines with lower ADA expression or activity (92.1, MM28, and Omm1), 160 µM cordycepin treatment showed significant anticancer effects, while growth in multiple lines with high levels of ADA was not inhibited. Consistent with the results in uveal melanoma, the therapeutic effects of cordycepin varied depending on ADA in other types of cancer as well. In retinoblastoma, AT/RT, and diffuse midline glioma H3 K27-altered cells with low ADA, cordycepin significantly inhibited cell growth and increased apoptotic markers, including cleaved PARP, while those with higher ADA expression were more resistant. Our examination of human uveal melanoma tissues showed that more than 90% of tumors had lower expression levels of ADA protein, suggesting that cordycepin treatment may be effective in a large proportion of cases. 

Combination therapy with cordycepin and ADA inhibitors was effective in treating a range of malignant tumors of the eye, brain, and pancreas. These are all tumors with extremely poor overall survival. Uveal melanoma is almost always fatal when it metastasizes to the liver or lungs [18], with no clinically proven therapies for metastatic disease [55,56,57,58]. Outcomes for the pediatric brain tumors we examined, AT/RT and diffuse midline glioma H3K27M mutant, as well as for pancreatic carcinoma, are similarly grim. The importance of ADA levels in predicting the response of a broad range of tumor cell lines to cordycepin suggested a combination therapy approach. Indeed, prior studies found that inhibiting ADA can sensitize trypanosomiasis models [21,59] to cordycepin treatment, and this was also investigated in a limited number of malignant peripheral nerve sheath tumor (MPNST) and leukemic lines [22,23,39]. However, we believe that our study is the first to test cordycepin in combination with ADA inhibitors in a broad range of tumor cell lines. 

Inhibition of ADA activity using siADA and small molecule ADA inhibitors greatly enhanced the anticancer effects of low cordycepin concentration (1–20 µM) in uveal melanoma cells, with a significant score in formal synergy testing. This included BAP1 mutant uveal melanoma lines (MM28, MP46, and MP38), a molecular change strongly associated with liver metastasis [60]. The combination approach was also successful in inhibiting the growth of MP46 xenografts with high ADA. These preclinical studies suggest that cordycepin may be effective in BAP1 mutant metastatic disease, for which no good therapies currently exist. Similar results were found in other highly aggressive tumor types. However, previous in vivo studies showed hepatic and renal toxicity [45]. Therefore, finding the specific range of ADA concentrations for combination treatment with limited toxicity is necessary, and this represents a limitation for the proposed combination therapy. 

A final exciting aspect of our work is the identification of Hsp90 as a novel target of cordycepin. Targeted therapies inhibiting the function of Hsp90 have been challenging to develop, partially due to toxicities, and no compounds have been approved for clinical work [61]. Validation of cordycepin as an Hsp90 inhibitor, therefore, has significant clinical potential. The structure of cordycepin is similar to the precursor of adenosine triphosphate (ATP); thus, we hypothesized that it could interact with the ATP binding pocket of Hsp90, potentially modulating its function. A computational docking model showed a high possibility that cordycepin could bind to Hsp90, and our ATP-agarose pull-down assay showed it competitively binds to the N-terminal ATP binding pocket of Hsp90. Furthermore, inhibition of Hsp90 by cordycepin leads to the degradation of client proteins, including EGFR, Akt, and ERK. Unlike some previously described Hsp90 inhibitors [62,63], cordycepin did not induce Hsp70 protein expression as a feedback effect. Since Cordycepin/3′-deoxyadenosine could also potentially be converted to 3′-deoxyadenosine triphosphate, we investigated whether 3′-deoxyadenosine or 3′-deoxyadenosine triphosphate would have a higher binding affinity to the ATP binding pocket. However, cordycepin triphosphate did not compete with ATP. Taken together, this represents a novel mechanism of action for cordycepin and one with potentially broad clinical applications. 

## 5. Conclusions

**Figure 7:**
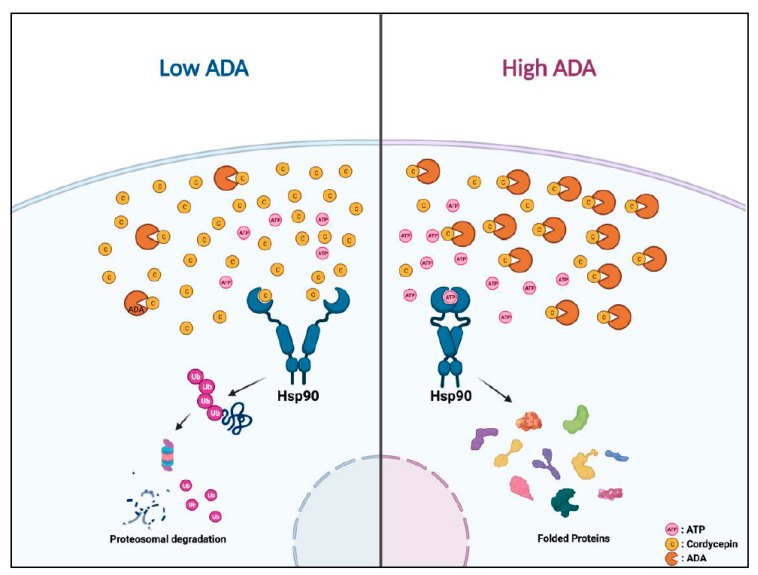
Graphical abstract for the action mechanism of cordycepin. Proposed mechanism of action.

Cordycepin slows migration, growth, and clonogenicity of uveal melanoma and other aggressive malignancies with low ADA levels, but all tumors tested could be sensitized through co-treatment with ADA inhibitors. This suggests that ADA may represent a predictive biomarker for cordycepin response and that tumors resistant to monotherapy may be sensitive to combination approaches. We also found a novel mechanism of action for cordycepin, inhibition of Hsp90, leading to degradation of client proteins in oncogenic signaling pathways (Figure 7). Several limitations apply to our study. Only limited in vivo testing was performed, and the effects of cordycepin-based therapies on the hematogenous dissemination of uveal melanoma will need to be evaluated, as well as on orthotopic xenograft growth for other tumor types. A more extensive evaluation of which Hsp90 client proteins represent key functional targets in each tumor type will also be important. However, this work provides an initial rationale for performing such studies.

## Data Availability

All data will be available from the corresponding author on request.

## Supplementary Materials

The following supporting information can be downloaded at: https://www.mdpi.com/article/10.3390/cancers14133122/s1, Figure S1: Cordycepin inhibits growth of uveal melanoma in an adenosine deaminase (ADA) dependent manner; Figure S2: Combination treatment with cordycepin and an ADA inhibitor enhances inhibition of anchorage independent-, dependent colony formation and migration abilities in uveal melanoma; Figure S3: Cordycepin binds Hsp90 and disrupts its function, resulting in degradation of client proteins; Figure S4: Antitumor effects of cordycepin in mice xenograft tumor models; Table S1: IC50 of Cordycepin in various cancer cells; File S1: Original Western blot figures.

## References

[1] Radhi M., Ashraf S., Lawrence S., Tranholm A.A., Wellham P.A.D., Hafeez A., Khamis A.S., Thomas R., McWilliams D., De Moor C.H. (2021). A Systematic Review of the Biological Effects of Cordycepin. Molecules.

[2] Cohen N., Cohen J., Asatiani M.D., Varshney V.K., Yu H.-T., Yang Y.-C., Li Y.-H., Mau J.-L., Wasser S.P. (2014). Chemical composition and nutritional and medicinal value of fruit bodies and submerged cultured mycelia of culinary-medicinal higher Basidiomycetes mushrooms. Int. J. Med. Mushrooms.

[3] Lee J.B., Radhi M., Cipolla E., Gandhi R.D., Sarmad S., Zgair A., Kim T.H., Feng W., Qin C., Adrower C. (2019). A novel nucleoside rescue metabolic pathway may be responsible for therapeutic effect of orally administered cordycepin. Sci. Rep..

[4] Nakamura K., Shinozuka K., Yoshikawa N. (2015). Anticancer and antimetastatic effects of cordycepin, an active component of Cordyceps sinensis. J. Pharmacol. Sci..

[5] Yao W.-L., Ko B.-S., Liu T.-A., Liang S.-M., Liu C.-C., Lu Y.-J., Tzean S.-S., Shen T.-L., Liou J.-Y. (2014). Cordycepin suppresses integrin/FAK signaling and epithelial-mesenchymal transition in hepatocellular carcinoma. Anti-Cancer Agents Med. Chem. (Former. Curr. Med. Chem.-Anti-Cancer Agents).

[6] Pao H.-Y., Pan B.-S., Leu S.-F., Huang B.-M. (2012). Cordycepin stimulated steroidogenesis in MA-10 mouse Leydig tumor cells through the protein kinase C Pathway. J. Agric. Food Chem..

[7] Chen Y., Yang S.-H., Hueng D.-Y., Syu J.-P., Liao C.-C., Wu Y.-C. (2014). Cordycepin induces apoptosis of C6 glioma cells through the adenosine 2A receptor-p53-caspase-7-PARP pathway. Chem.-Biol. Interact..

[8] Cao H.-L., Liu Z.-J., Chang Z. (2017). Cordycepin induces apoptosis in human bladder cancer cells via activation of A3 adenosine receptors. Tumor Biol..

[9] Nakamura K., Yoshikawa N., Yamaguchi Y., Kagota S., Shinozuka K., Kunitomo M. (2006). Antitumor effect of cordycepin (3′-deoxyadenosine) on mouse melanoma and lung carcinoma cells involves adenosine A3 receptor stimulation. Anticancer Res..

[10] Yoshikawa N., Yamada S., Takeuchi C., Kagota S., Shinozuka K., Kunitomo M., Nakamura K. (2008). Cordycepin (3′-deoxyadenosine) inhibits the growth of B16-BL6 mouse melanoma cells through the stimulation of adenosine A 3 receptor followed by glycogen synthase kinase-3β activation and cyclin D 1 suppression. Naunyn-Schmiedeberg’s Arch. Pharmacol..

[11] Wang Z., Wu X., Liang Y.-N., Wang L., Song Z.-X., Liu J.-L., Tang Z.-S. (2016). Cordycepin induces apoptosis and inhibits proliferation of human lung cancer cell line H1975 via inhibiting the phosphorylation of EGFR. Molecules.

[12] Van Raamsdonk C.D., Griewank K.G., Crosby M.B., Garrido M.C., Vemula S., Wiesner T., Obenauf A.C., Wackernagel W., Green G., Bouvier N. (2010). Mutations in GNA11 in uveal melanoma. N. Engl. J. Med..

[13] Onken M.D., Worley L.A., Long M.D., Duan S., Council M.L., Bowcock A.M., Harbour J.W. (2008). Oncogenic mutations in GNAQ occur early in uveal melanoma. Investig. Ophthalmol. Vis. Sci..

[14] Davies B.R., Logie A., McKay J.S., Martin P., Steele S., Jenkins R., Cockerill M., Cartlidge S., Smith P.D. (2007). AZD6244 (ARRY-142886), a potent inhibitor of mitogen-activated protein kinase/extracellular signal-regulated kinase kinase 1/2 kinases: Mechanism of action in vivo, pharmacokinetic/pharmacodynamic relationship, and potential for combination in preclinical models. Mol. Cancer Ther..

[15] Harbour J.W., Onken M.D., Roberson E.D., Duan S., Cao L., Worley L.A., Council M.L., Matatall K.A., Helms C., Bowcock A.M. (2010). Frequent mutation of BAP1 in metastasizing uveal melanomas. Science.

[16] Harbour J.W. (2012). The genetics of uveal melanoma: An emerging framework for targeted therapy. Pigment Cell Melanoma Res..

[17] Landreville S., Agapova O.A., Matatall K.A., Kneass Z.T., Onken M.D., Lee R.S., Bowcock A.M., Harbour J.W. (2012). Histone deacetylase inhibitors induce growth arrest and differentiation in uveal melanoma. Clin. Cancer Res..

[18] Singh A.D., Turell M.E., Topham A.K. (2011). Uveal melanoma: Trends in incidence, treatment, and survival. Ophthalmology.

[19] Flinn A.M., Gennery A.R. (2018). Adenosine deaminase deficiency: A review. Orphanet J. Rare Dis..

[20] Blackburn M.R., Kellems R.E. (2005). Adenosine deaminase deficiency: Metabolic basis of immune deficiency and pulmonary inflammation. Adv. Immunol..

[21] Rottenberg M.E., Masocha W., Ferella M., Petitto-Assis F., Goto H., Kristensson K., McCaffrey R., Wigzell H. (2005). Treatment of African trypanosomiasis with cordycepin and adenosine deaminase inhibitors in a mouse model. J. Infect. Dis..

[22] Koc Y., Urbano A.G., Sweeney E.B., McCaffrey R. (1996). Induction of apoptosis by cordycepin in ADA-inhibited TdT-positive leukemia cells. Leukemia.

[23] Lee M.-J., Lee J.-C., Hsieh J.-H., Lin M.-Y., Shih I.-A., You H.-L., Wang K. (2021). Cordycepin inhibits the proliferation of malignant peripheral nerve sheath tumor cells through the p53/Sp1/tubulin pathway. Am. J. Cancer Res..

[24] Taylor I.C., Hütt-Cabezas M., Brandt W.D., Kambhampati M., Nazarian J., Chang H.T., Warren K.E., Eberhart C.G., Raabe E.H. (2015). Disrupting NOTCH slows diffuse intrinsic pontine glioma growth, enhances radiation sensitivity, and shows combinatorial efficacy with bromodomain inhibition. J. Neuropathol. Exp. Neurol..

[25] Asnaghi L., Handa J.T., Merbs S.L., Harbour J.W., Eberhart C.G. (2013). A role for Jag2 in promoting uveal melanoma dissemination and growth. Investig. Ophthalmol. Vis. Sci..

[26] Asnaghi L., Ebrahimi K.B., Schreck K.C., Bar E.E., Coonfield M.L., Bell W.R., Handa J., Merbs S.L., Harbour J.W., Eberhart C.G. (2012). Notch signaling promotes growth and invasion in uveal melanoma. Clin. Cancer Res..

[27] Ianevski A., Giri A.K., Aittokallio T. (2020). SynergyFinder 2.0: Visual analytics of multi-drug combination synergies. Nucleic Acids Res..

[28] Lewis H.D., Leveridge M., Strack P.R., Haldon C.D., O’Neil J., Kim H., Madin A., Hannam J.C., Look A.T., Kohl N. (2007). Apoptosis in T cell acute lymphoblastic leukemia cells after cell cycle arrest induced by pharmacological inhibition of notch signaling. Chem. Biol..

[29] Parr R.G. (1980). Density functional theory of atoms and molecules. Horizons of Quantum Chemistry.

[30] Lee C., Yang W., Parr R.G. (1988). Development of the Colle-Salvetti correlation-energy formula into a functional of the electron density. Phys. Rev. B.

[31] Grimme S., Antony J., Ehrlich S., Krieg H. (2010). A consistent and accurate ab initio parametrization of density functional dispersion correction (DFT-D) for the 94 elements H-Pu. J. Chem. Phys..

[32] Becke A. (1988). Density-functional exchange-energy approximation with correct asymptotic behavior. Phys. Rev. A.

[33] Hay P.J., Wadt W.R. (1985). Ab initio effective core potentials for molecular calculations. Potentials for the transition metal atoms Sc to Hg. J. Chem. Phys..

[34] Dunning Jr T.H. (1989). Gaussian basis sets for use in correlated molecular calculations. I. The atoms boron through neon and hydrogen. J. Chem. Phys..

[35] Edinger S.R., Cortis C., Shenkin P.S., Friesner R.A. (1997). Solvation free energies of peptides: Comparison of approximate continuum solvation models with accurate solution of the Poisson–Boltzmann equation. J. Phys. Chem. B.

[36] Friedrichs M., Zhou R., Edinger S.R., Friesner R.A. (1999). Poisson−Boltzmann analytical gradients for molecular modeling calculations. J. Phys. Chem. B.

[37] Marten B., Kim K., Cortis C., Friesner R.A., Murphy R.B., Ringnalda M.N., Sitkoff D., Honig B. (1996). New model for calculation of solvation free energies: Correction of self-consistent reaction field continuum dielectric theory for short-range hydrogen-bonding effects. J. Phys. Chem..

[38] Cristalli G., Costanzi S., Lambertucci C., Lupidi G., Vittori S., Volpini R., Camaioni E. (2001). Adenosine deaminase: Functional implications and different classes of inhibitors. Med. Res. Rev..

[39] Li G., Nakagome I., Hirono S., Itoh T., Fujiwara R. (2015). Inhibition of adenosine deaminase (ADA)-mediated metabolism of cordycepin by natural substances. Pharmacol. Res. Perspect..

[40] Lee E.J., Kim W.J., Moon S.K. (2010). Cordycepin suppresses TNF-alpha-induced invasion, migration and matrix metalloproteinase-9 expression in human bladder cancer cells. Phytother. Res..

[41] Kane B.J., Kuhn J.G., Roush M.K. (1992). Pentostatin: An adenosine deaminase inhibitor for the treatment of hairy cell leukemia. Ann. Pharmacother..

[42] Lloyd H., Fredholm B. (1995). Involvement of adenosine deaminase and adenosine kinase in regulating extracellular adenosine concentration in rat hippocampal slices. Neurochem. Int..

[43] Tesch A.M., MacDonald M.H., Kollias-Baker C., Benton H.P. (2002). Chondrocytes respond to adenosine via A2receptors and activity is potentiated by an adenosine deaminase inhibitor and a phosphodiesterase inhibitor. Osteoarthr. Cartil..

[44] Hsu P.-Y., Lin Y.-H., Yeh E.-L., Lo H.-C., Hsu T.-H., Su C.-C. (2017). Cordycepin and a preparation from Cordyceps militaris inhibit malignant transformation and proliferation by decreasing EGFR and IL-17RA signaling in a murine oral cancer model. Oncotarget.

[45] Schwenzer H., De Zan E., Elshani M., Van Stiphout R., Kudsy M., Morris J., Ferrari V., Um I.H., Chettle J., Kazmi F. (2021). The Novel Nucleoside Analogue ProTide NUC-7738 Overcomes Cancer Resistance Mechanisms In Vitro and in a First-in-Human Phase I Clinical Trial. Clin. Cancer Res..

[46] Liu Y.V., Semenza G.L. (2007). RACK1 vs. HSP90: Competition for HIF-1α degradation vs. stabilization. Cell Cycle.

[47] Kim W.-Y., Oh S.H., Woo J.-K., Hong W.K., Lee H.-Y. (2009). Targeting heat shock protein 90 overrides the resistance of lung cancer cells by blocking radiation-induced stabilization of hypoxia-inducible factor-1α. Cancer Res..

[48] Oh S.H., Woo J.K., Yazici Y.D., Myers J.N., Kim W.-Y., Jin Q., Hong S.S., Park H.-J., Suh Y.-G., Kim K.-W. (2007). Structural basis for depletion of heat shock protein 90 client proteins by deguelin. J. Natl. Cancer Inst..

[49] Lee S.-C., Min H.-Y., Choi H., Kim H.S., Kim K.-C., Park S.-J., Seong M.A., Seo J.H., Park H.-J., Suh Y.-G. (2015). Synthesis and evaluation of a novel deguelin derivative, L80, which disrupts ATP binding to the C-terminal domain of heat shock protein 90. Mol. Pharmacol..

[50] Lee S.-C., Min H.-Y., Choi H., Bae S.Y., Park K.H., Hyun S.Y., Lee H.J., Moon J., Park S.-H., Kim J.Y. (2016). Deguelin analogue SH-1242 inhibits Hsp90 activity and exerts potent anticancer efficacy with limited neurotoxicity. Cancer Res..

[51] Kudryavtsev V.A., Khokhlova A.V., Mosina V.A., Selivanova E.I., Kabakov A.E. (2017). Induction of Hsp70 in tumor cells treated with inhibitors of the Hsp90 activity: A predictive marker and promising target for radiosensitization. PLoS ONE.

[52] Tassone G., Mangani S., Botta M., Pozzi C. (2018). Probing the role of Arg97 in Heat shock protein 90 N-terminal domain from the parasite Leishmania braziliensis through site-directed mutagenesis on the human counterpart. Biochim. Biophys. Acta (BBA)-Proteins Proteom..

[53] Obermann W.M., Sondermann H., Russo A.A., Pavletich N.P., Hartl F.U. (1998). In Vivo function of Hsp90 is dependent on ATP binding and ATP hydrolysis. J. Cell Biol..

[54] Sanchez-Martin C., Serapian S.A., Colombo G., Rasola A. (2020). Dynamically shaping chaperones. Allosteric modulators of HSP90 family as regulatory tools of cell metabolism in neoplastic progression. Front. Oncol..

[55] Yang J., Manson D.K., Marr B.P., Carvajal R.D. (2018). Treatment of uveal melanoma: Where are we now?. Ther. Adv. Med. Oncol..

[56] Tsai K.K., Bollin K.B., Patel S.P. (2018). Obstacles to improving outcomes in the treatment of uveal melanoma. Cancer.

[57] Park J.J., Diefenbach R.J., Joshua A.M., Kefford R.F., Carlino M.S., Rizos H. (2018). Oncogenic signaling in uveal melanoma. Pigment Cell Melanoma Res..

[58] Rantala E.S., Hernberg M., Kivela T.T. (2019). Overall survival after treatment for metastatic uveal melanoma: A systematic review and meta-analysis. Melanoma Res..

[59] Da Silva A.S., Wolkmer P., Nunes J.T., Duck M.R., Oliveira C.B., Gressler L.T., Costa M.M., Zanette R.A., Mazzanti C.M., Lopes S.T. (2011). Susceptibility of Trypanosoma evansi to cordycepin. Biomed. Pharmacother..

[60] Kalirai H., Dodson A., Faqir S., Damato B., Coupland S. (2014). Lack of BAP1 protein expression in uveal melanoma is associated with increased metastatic risk and has utility in routine prognostic testing. Br. J. Cancer.

[61] Yuno A., Lee M.J., Lee S., Tomita Y., Rekhtman D., Moore B., Trepel J.B. (2018). Clinical Evaluation and Biomarker Profiling of Hsp90 Inhibitors. Methods Mol. Biol..

[62] Ma X., Hibbert B., McNulty M., Hu T., Zhao X., Ramirez F.D., Simard T., de Belleroche J.S., O’Brien E.R. (2014). Heat shock protein 27 attenuates neointima formation and accelerates reendothelialization after arterial injury and stent implantation: Importance of vascular endothelial growth factor up-regulation. FASEB J..

[63] Erlichman C. (2009). Tanespimycin: The opportunities and challenges of targeting heat shock protein 90. Expert Opin. Investig. Drugs.

