# Supplementary material for: Cordycepin (3′-Deoxyadenosine) Suppresses Heat Shock Protein 90 Function and Targets Tumor Growth in an Adenosine Deaminase-Dependent Manner
Source: Cancers (Basel). 2022 Jun 25;14(13):3122. doi: 10.3390/cancers14133122 (PMC9264932; doi:10.3390/cancers14133122)

Figure 1.

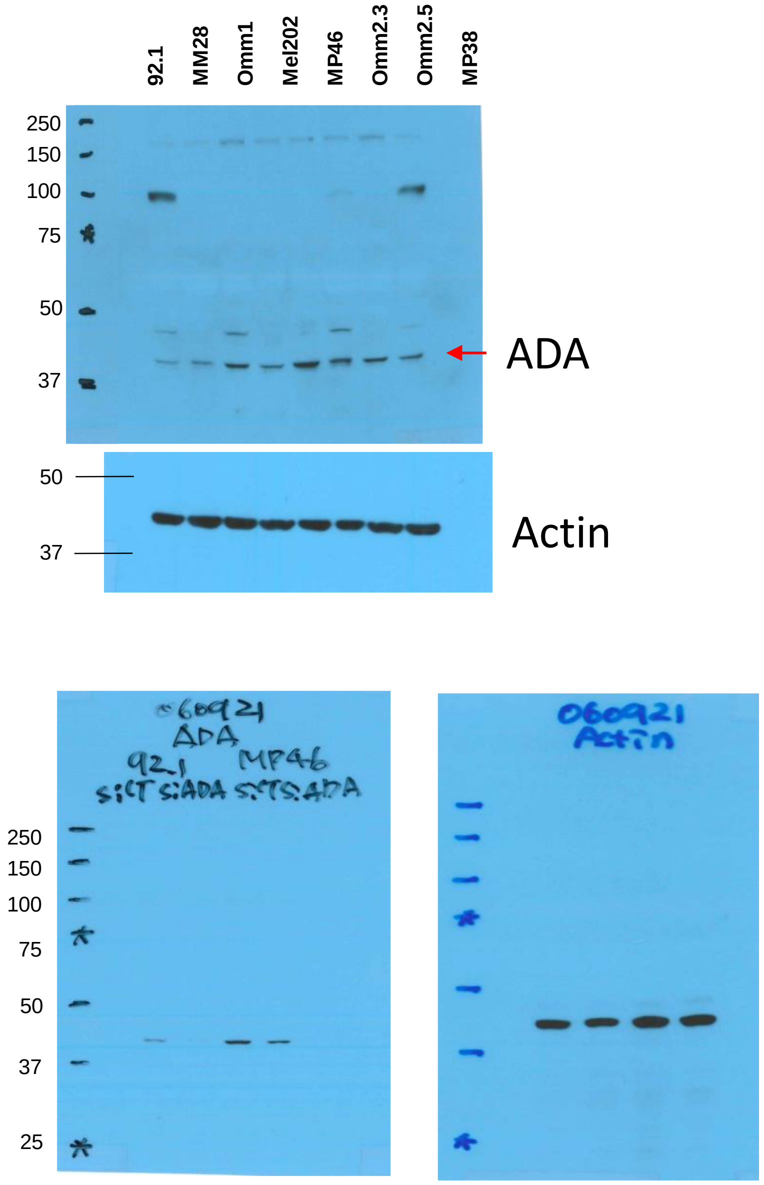
92.1
MM28
Omm1
Mel202
MP46
Omm2.3
Omm2.5
MP38
250
150
100
75
50
37
ADA
50
37
Actin
060921
ADA
92.1
MP46
250
150
100
75
50
37
25
060921
Actin

Figure 4.

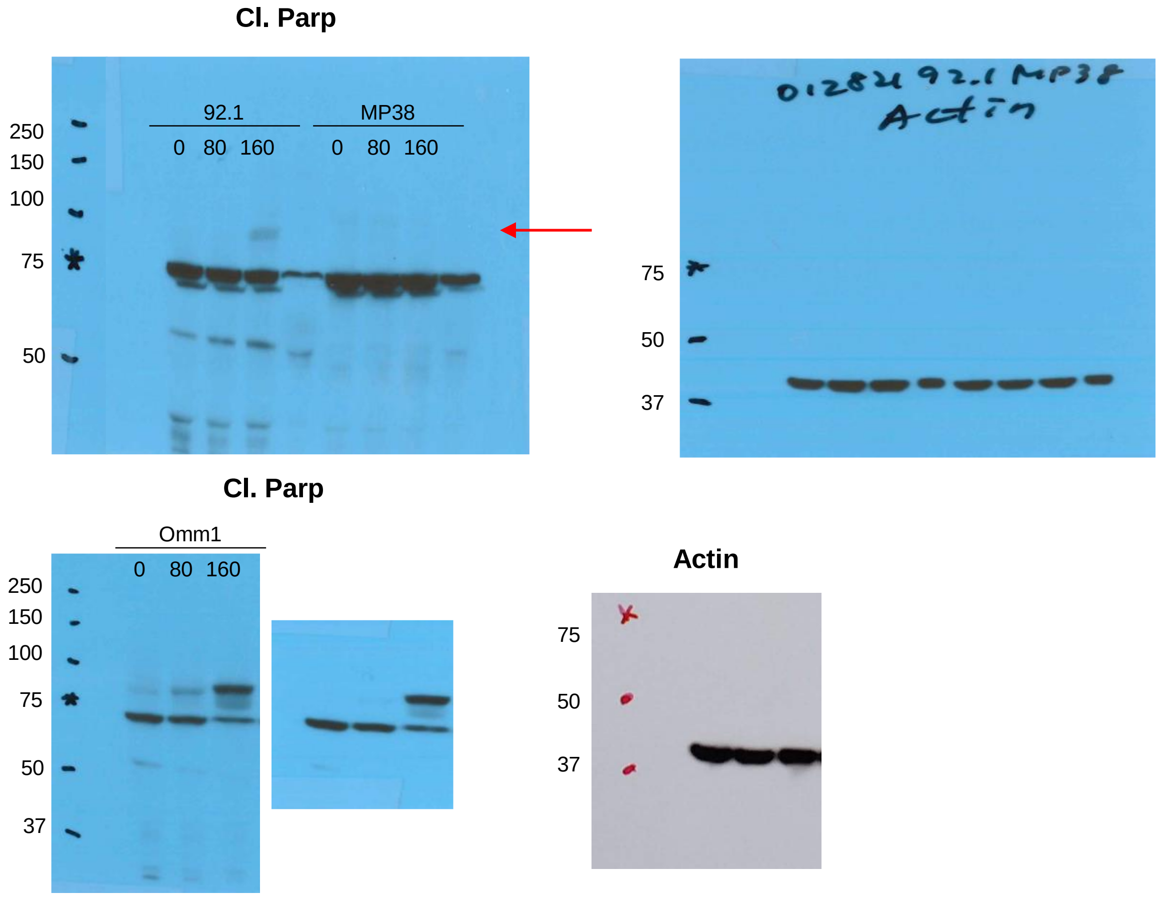
Cl. Parp
92.1
MP38
0 80 160
0 80 160
250
150
100
75
50
012824 92.1 MP38
Actin
75
50
37
Cl. Parp
Omm1
0 80 160
250
150
100
75
50
37
Actin
75
50
37

Figure 4.

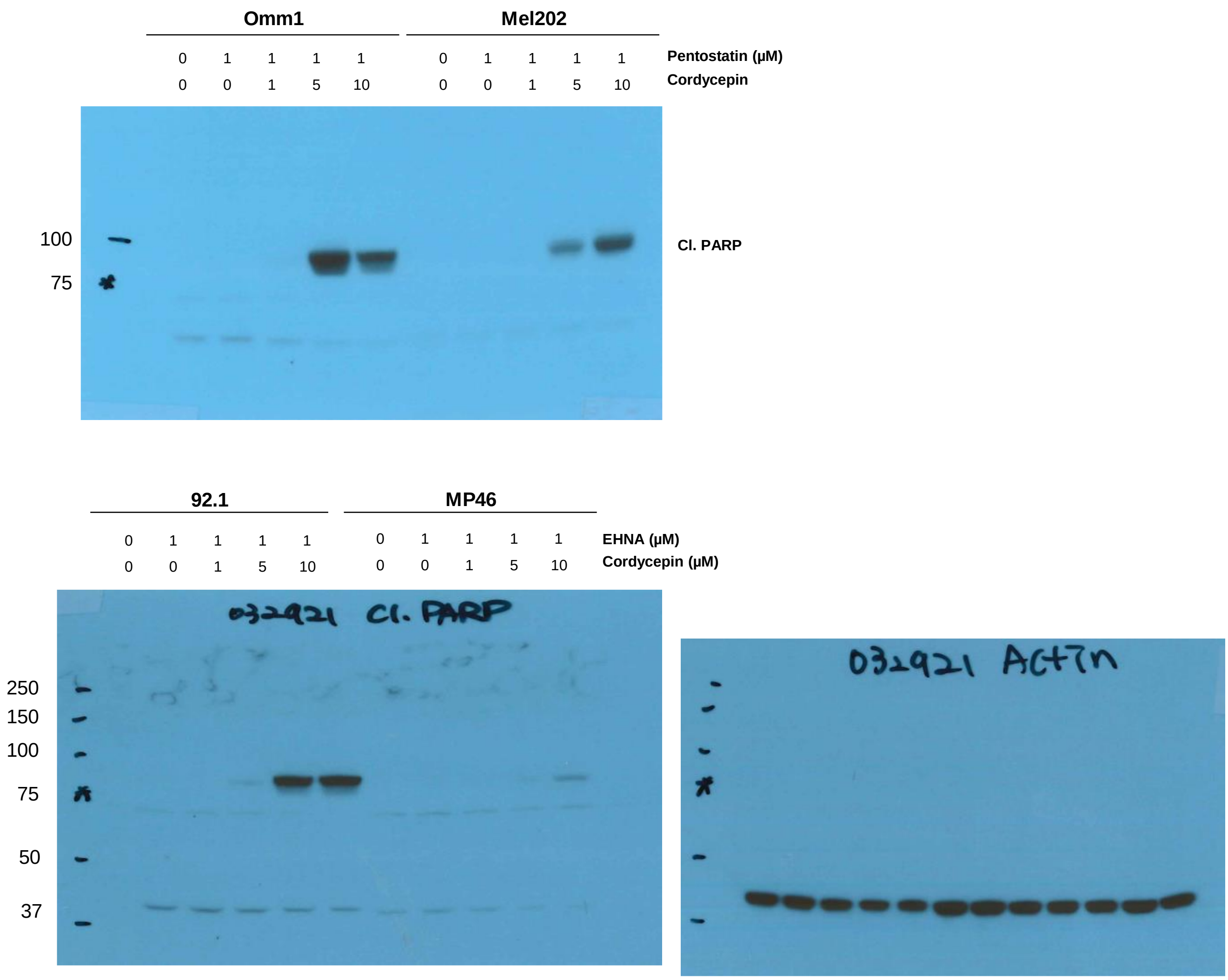
Omm1
Mel202
0 1 1 1 1 0 1 1 1 1
Pentostatin (μM)
0 0 1 5 10 0 0 1 5 10
Cordycepin
100
75
Cl. PARP
92.1
MP46
0 1 1 1 1 0 1 1 1 1
EHNA (μM)
0 0 1 5 10 0 0 1 5 10
Cordycepin (μM)
032921 Cl. PARP
250
150
100
75
50
37
032921 Actin

Figure 5.

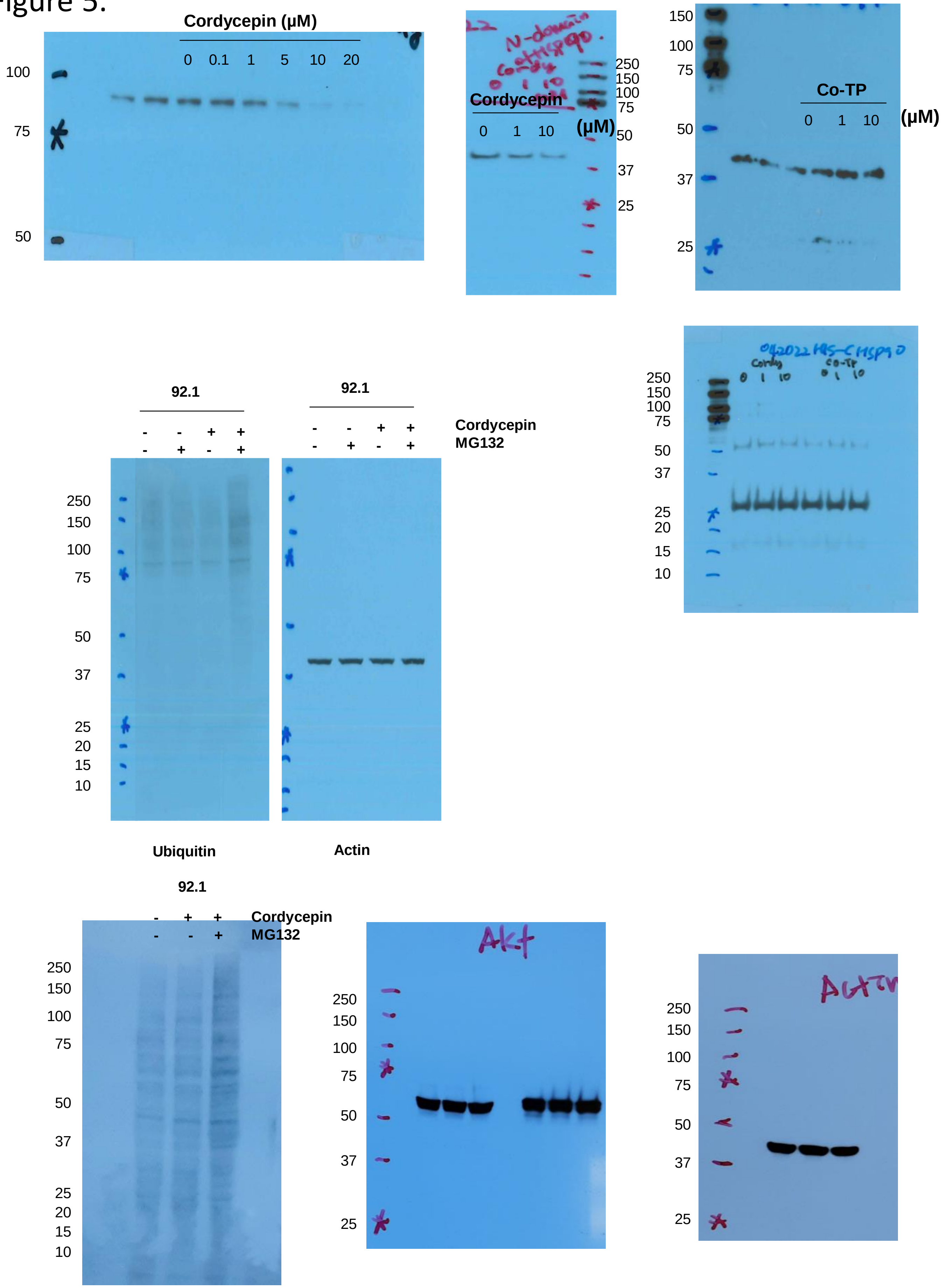
Cordycepin (µM)
0
0.1
1
5
10
20
100
75
50
Cordycepin
0
1
10
(µM)
250
150
100
75
50
37
25
Co-TP
0
1
10
(µM)
150
100
75
50
37
25
92.1
92.1
Cordycepin
MG132
250
150
100
75
50
37
25
20
15
10
Ubiquitin
Actin
92.1
Cordycepin
MG132
Akt

Figure 5.

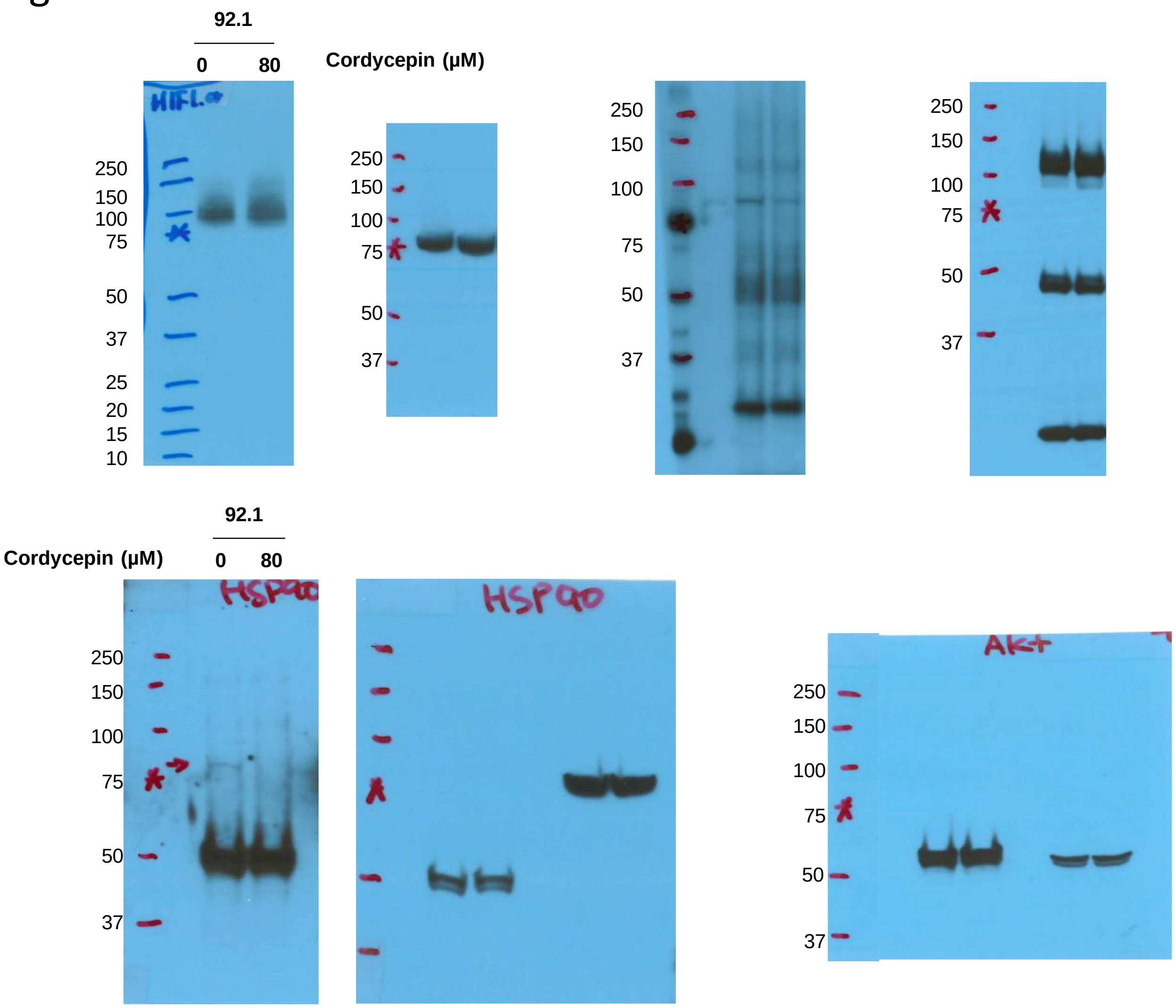
92.1
0
80
Cordycepin (μM)
250
150
100
75
50
37
25
20
15
10
250
150
100
75
50
37
250
150
100
75
50
37
250
150
100
75
50
37
92.1
Cordycepin (μM)
0
80
HSP90
250
150
100
75
50
37
HSP90
Akt
250
150
100
75
50
37

Figure 5.

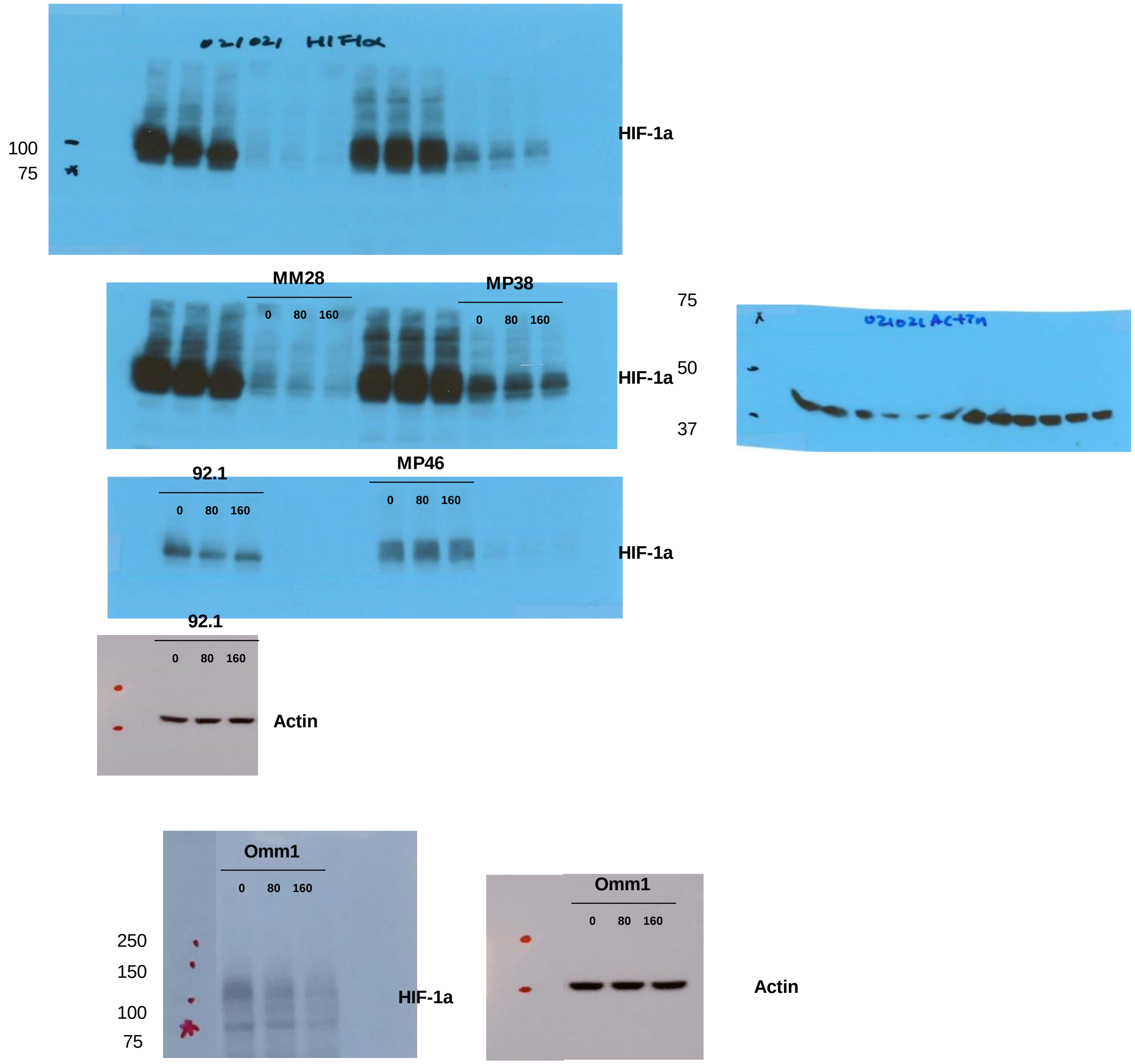
021021 HIF1α
100
75
HIF-1a
MM28
0 80 160
MP38
0 80 160
HIF-1a
75
50
37
021021 Actin
92.1
0 80 160
MP46
0 80 160
HIF-1a
92.1
0 80 160
Actin
Omm1
0 80 160
250
150
100
75
HIF-1a
Omm1
0 80 160
Actin

Figure 5.

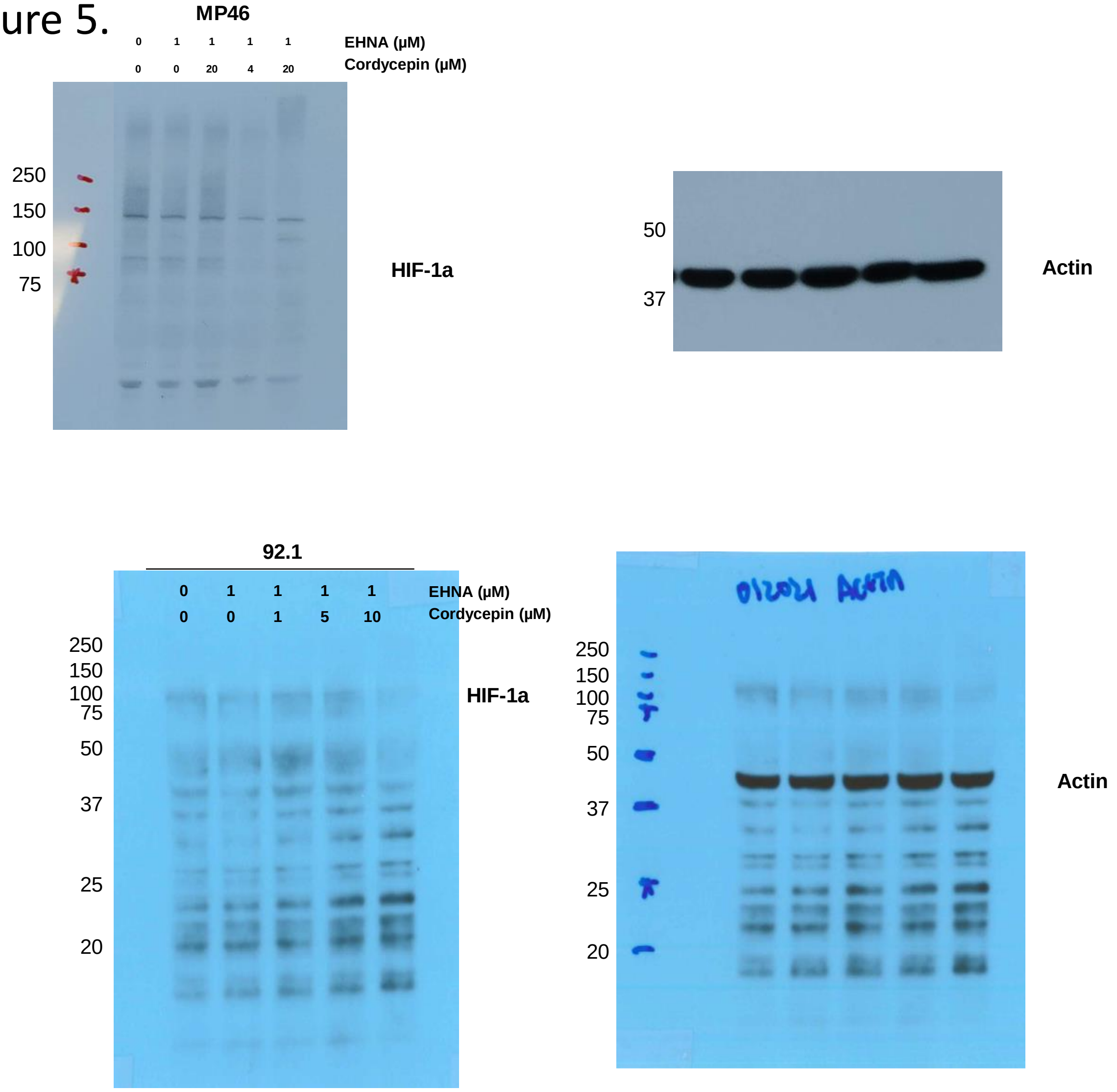
MP46
0 1 1 1 1 EHNA (μM)
0 0 20 4 20 Cordycepin (μM)
250
150
100
75
HIF-1a
50
37
Actin
92.1
0 1 1 1 1 EHNA (μM)
0 0 1 5 10 Cordycepin (μM)
250
150
100
75
50
37
25
20
HIF-1a
250
150
100
75
50
37
25
20
Actin

Figure 5.

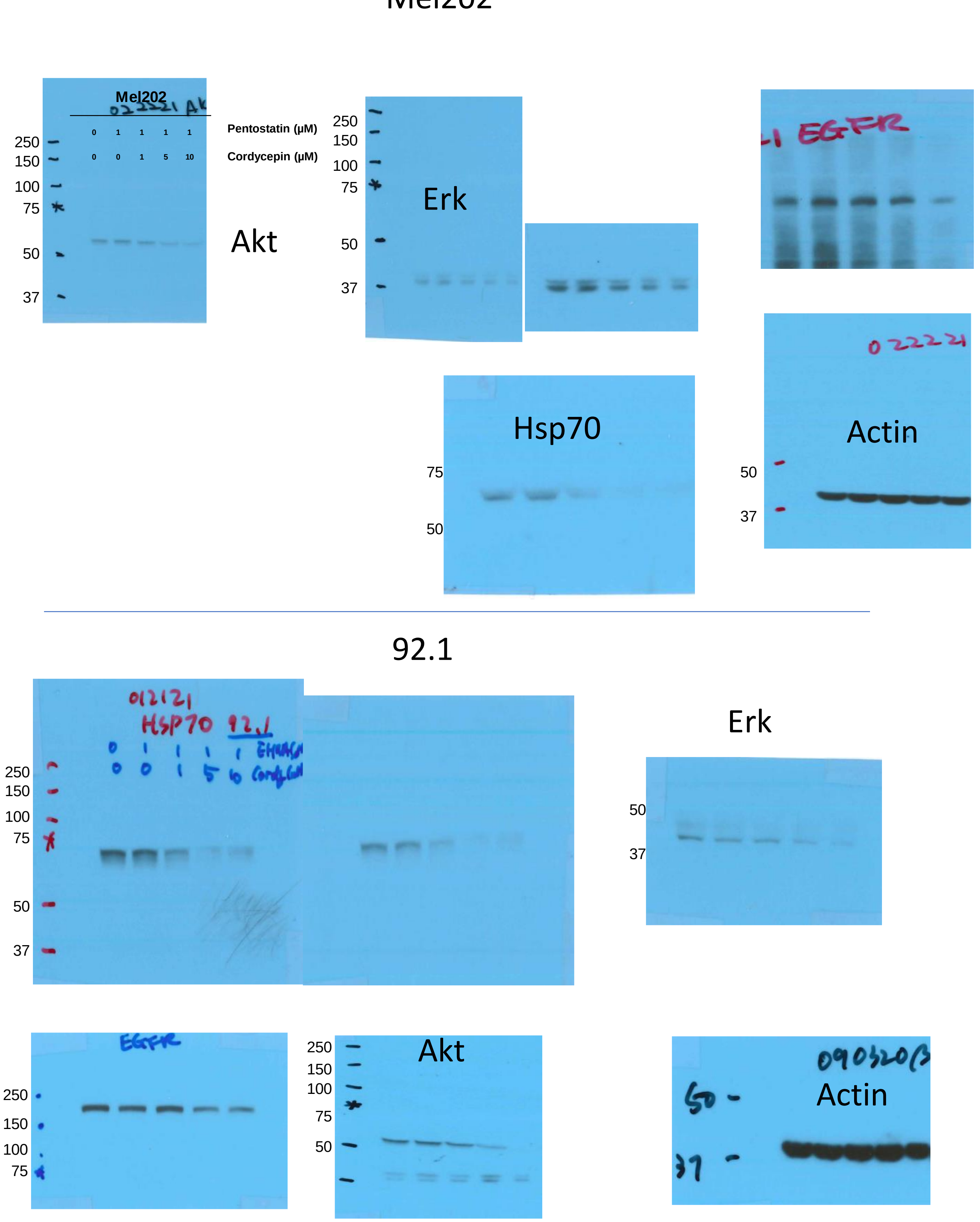

Mel202
Mel202
Pentostatin (µM)
0 1 1 1 1
Cordycepin (µM)
0 0 1 5 10
250
150
100
75
50
37
Akt
Erk
EGFR
Hsp70
Actin
92.1
Erk
EGFR
Akt
Actin

Figure 5.

MP38

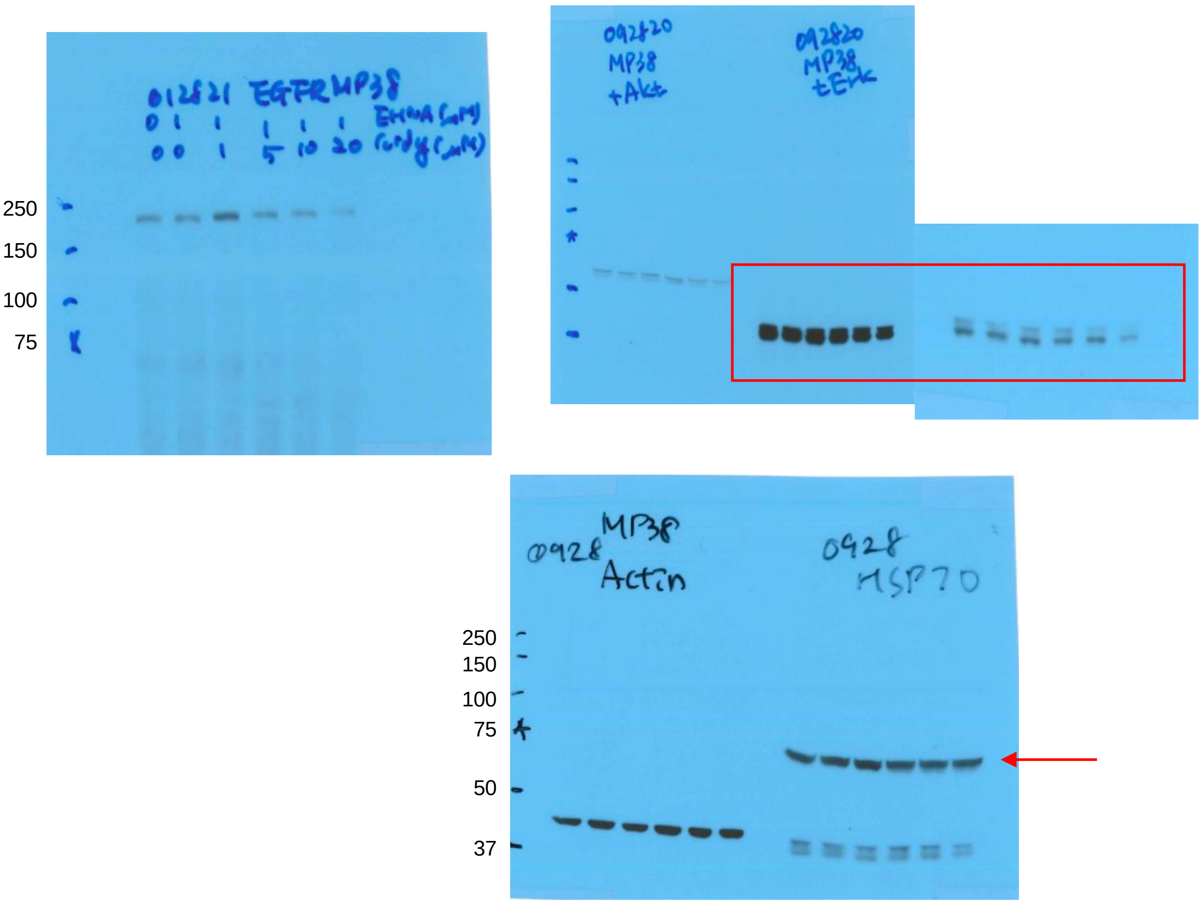

Figure 5.

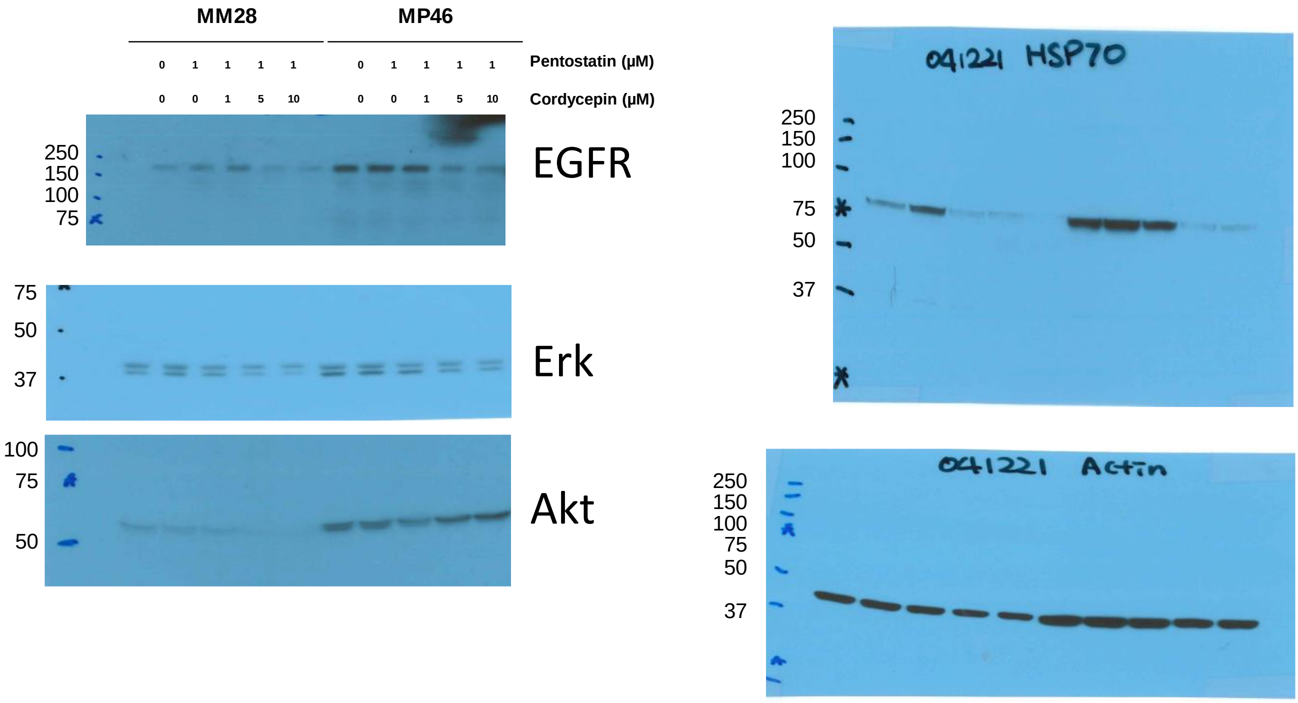
MM28
MP46
0 1 1 1 1 0 1 1 1 1
Pentostatin (µM)
0 0 1 5 10 0 0 1 5 10
Cordycepin (µM)
250
150
100
75
EGFR
75
50
37
Erk
100
75
50
Akt
041221 HSP70
250
150
100
75
50
37
041221 Actin
250
150
100
75
50
37

Figure 6.

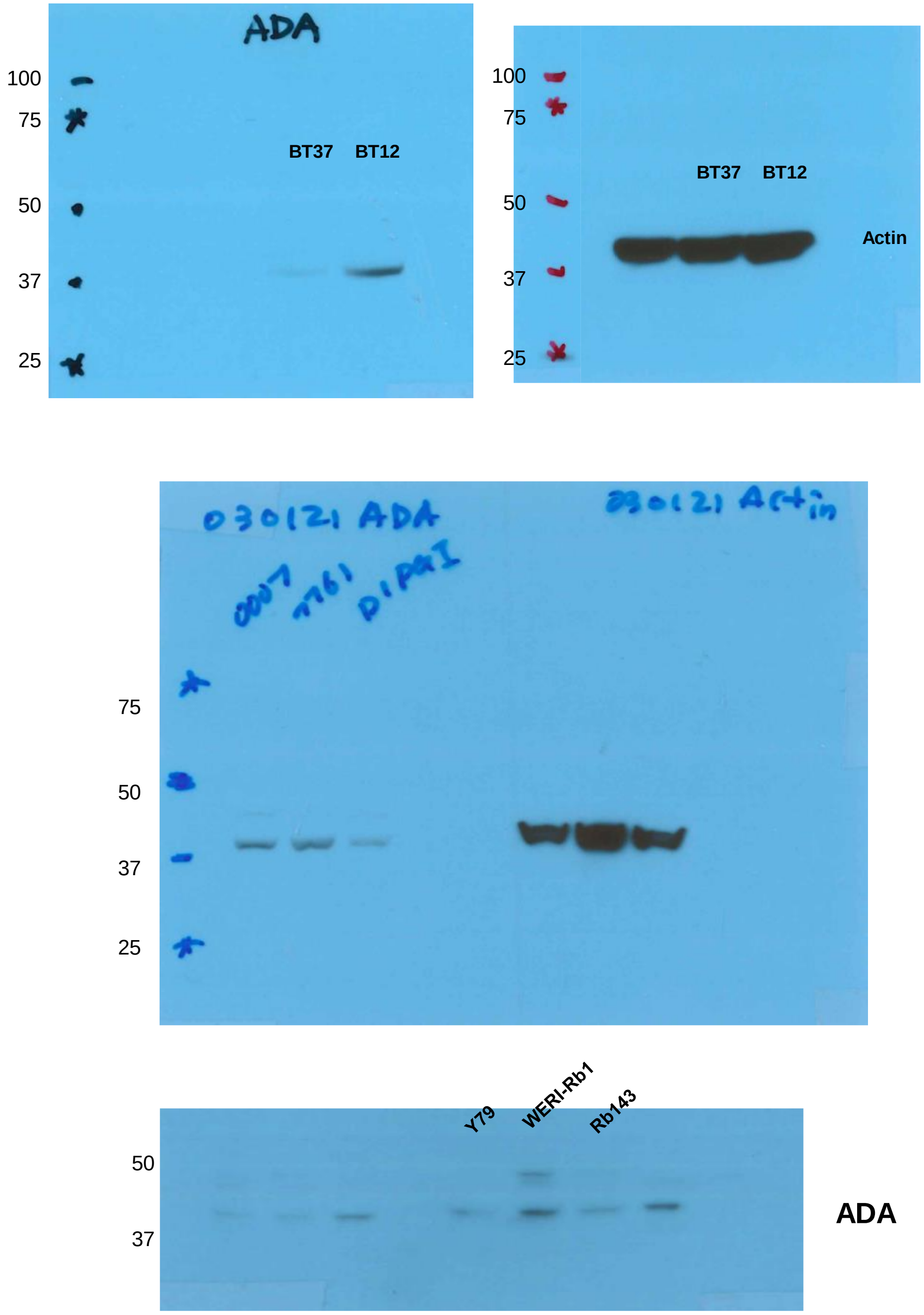
ADA
100
75
50
37
25
BT37
BT12
100
75
50
37
25
BT37
BT12
Actin
75
50
37
25
Y79
WERI-Rb1
Rb143
50
37
ADA

Figure 6.

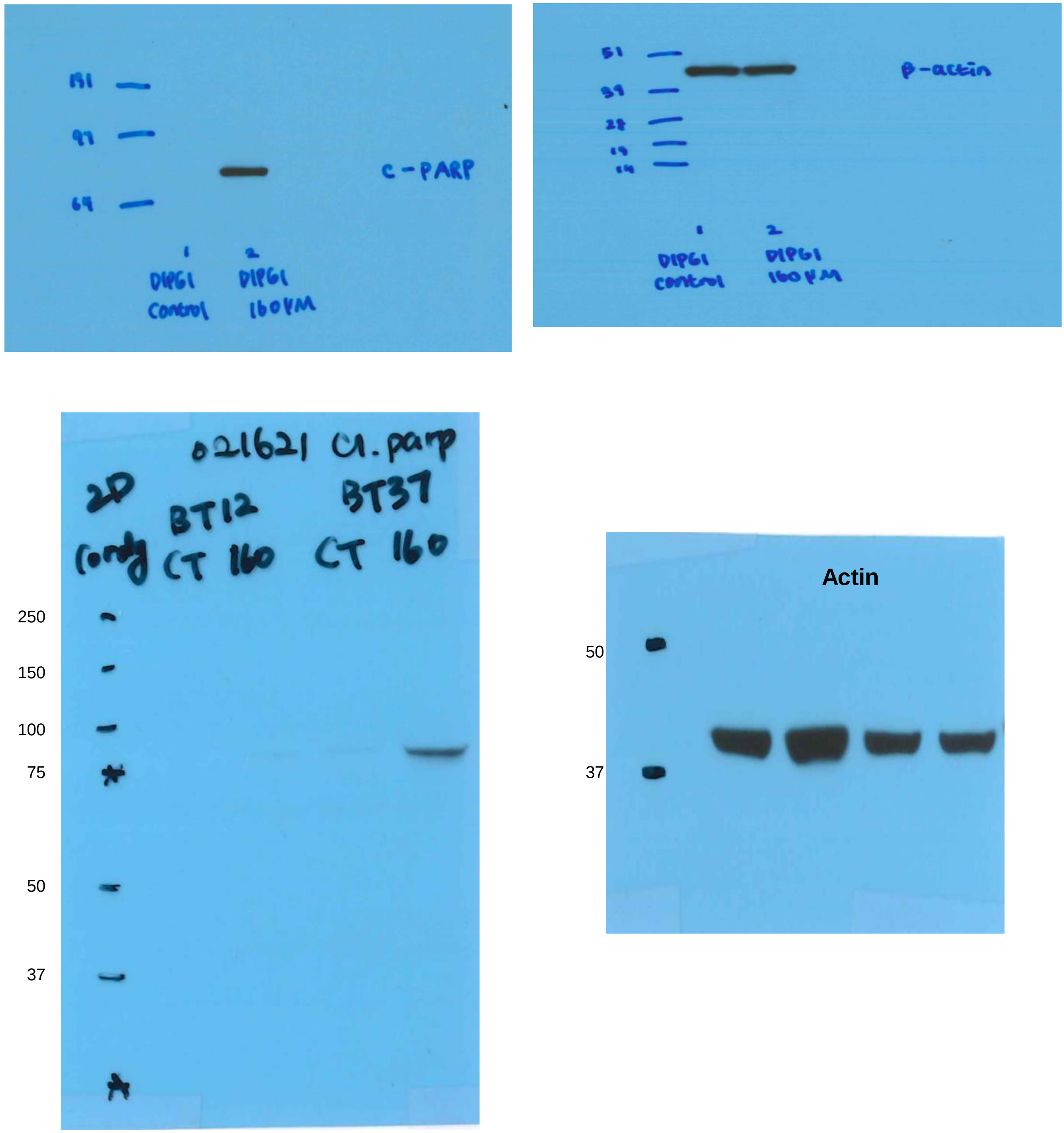

131
97
64
C-PARP
1
2
DIPG1
Control
DIPG1
160 μM
51
39
28
19
14
β-actin
1
2
DIPG1
control
DIPG1
160 μM
021621 Cl. parp
2D
Cond
BT12
CT 160
BT37
CT 160
250
150
100
75
50
37
Actin
50
37

Figure S3

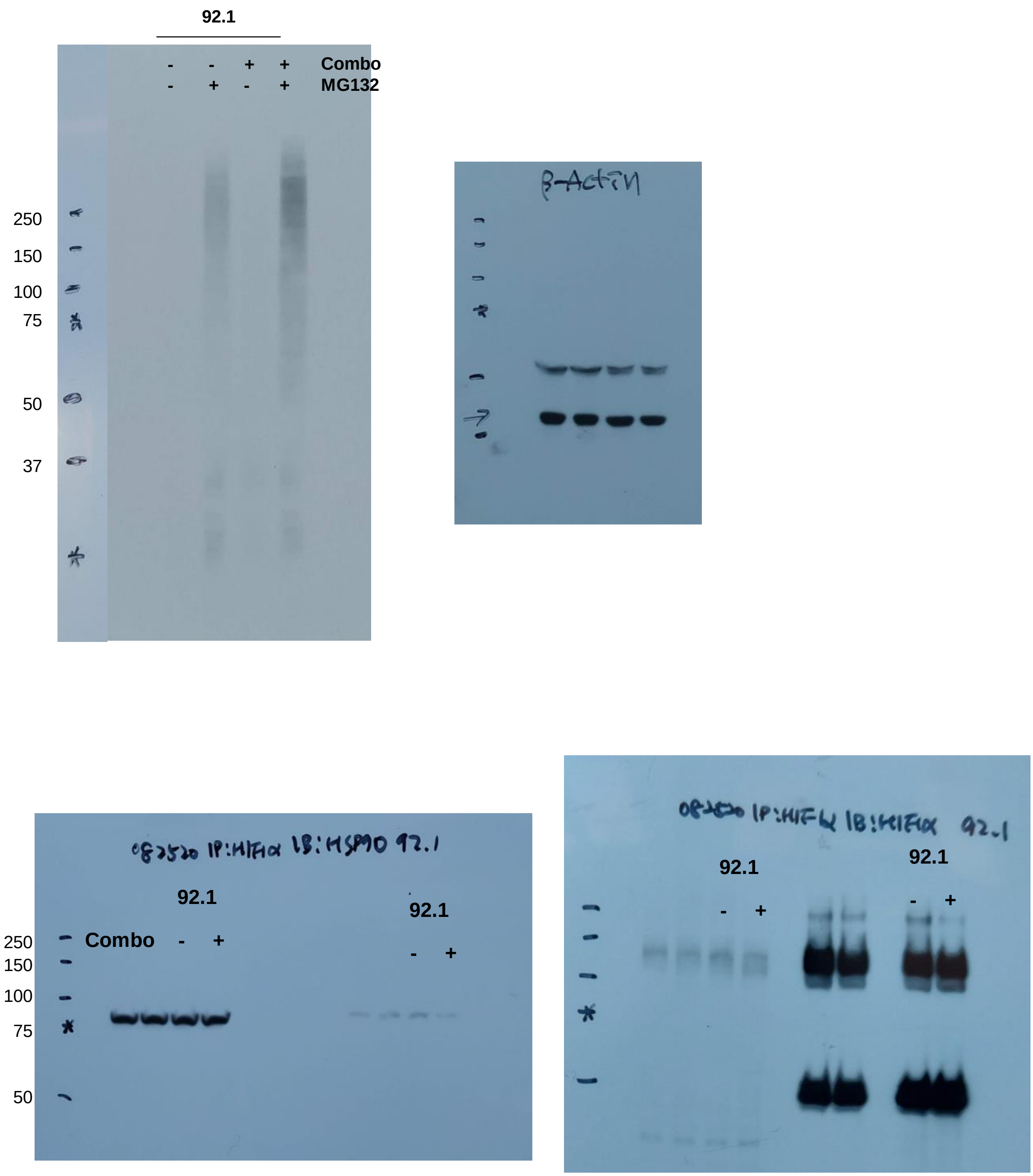

92.1
- - + + Combo
- + - + MG132
250
150
100
75
50
37
β-Actin
082520 IP:HIFα IB:HSP90 92.1
92.1
Combo - +
92.1
- +
250
150
100
75
50
082520 IP:HIFα IB:HIFα 92.1
92.1
- +
92.1
- +

Figure S3

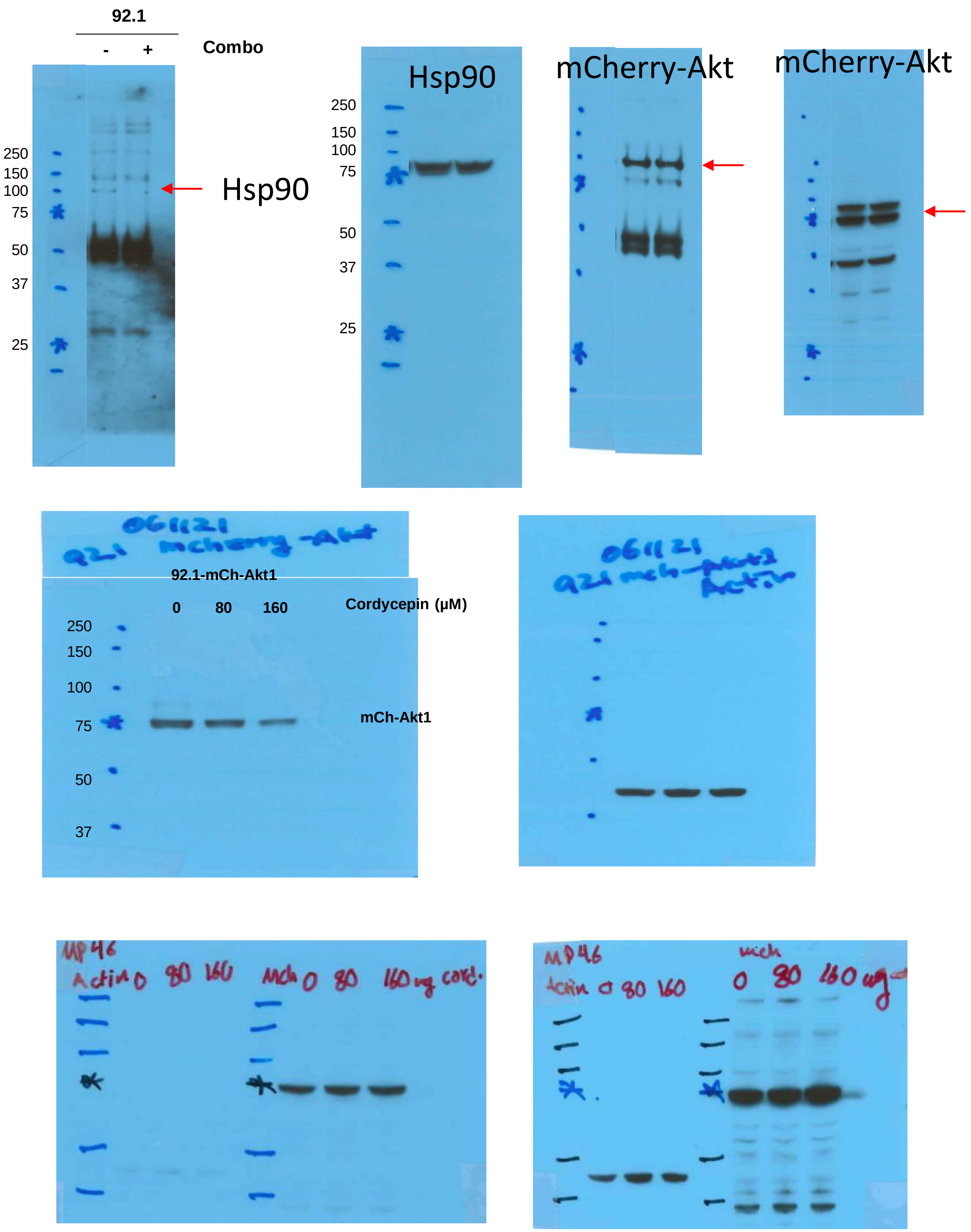
92.1
Combo
- +
250
150
100
75
50
37
25
Hsp90
Hsp90
250
150
100
75
50
37
25
mCherry-Akt
mCherry-Akt
92.1-mCh-Akt1
0 80 160
Cordycepin (μM)
250
150
100
75
50
37
mCh-Akt1

Figure S3

MM28

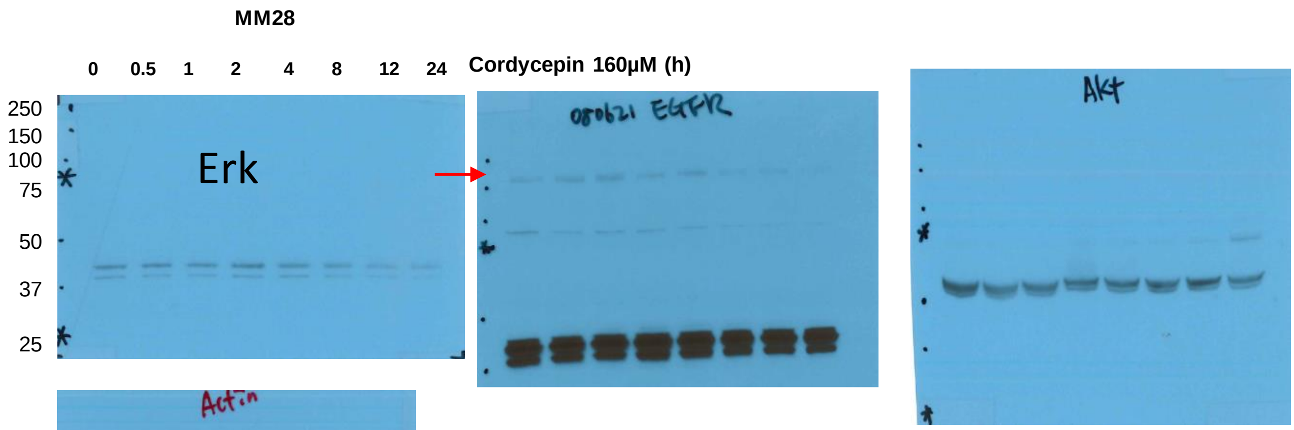

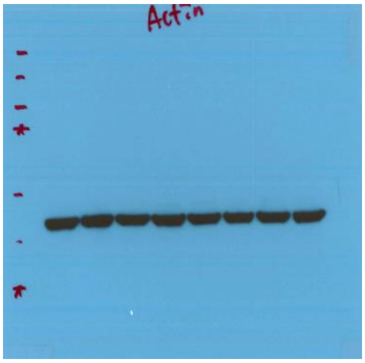

sF3C 92.1 and MP46

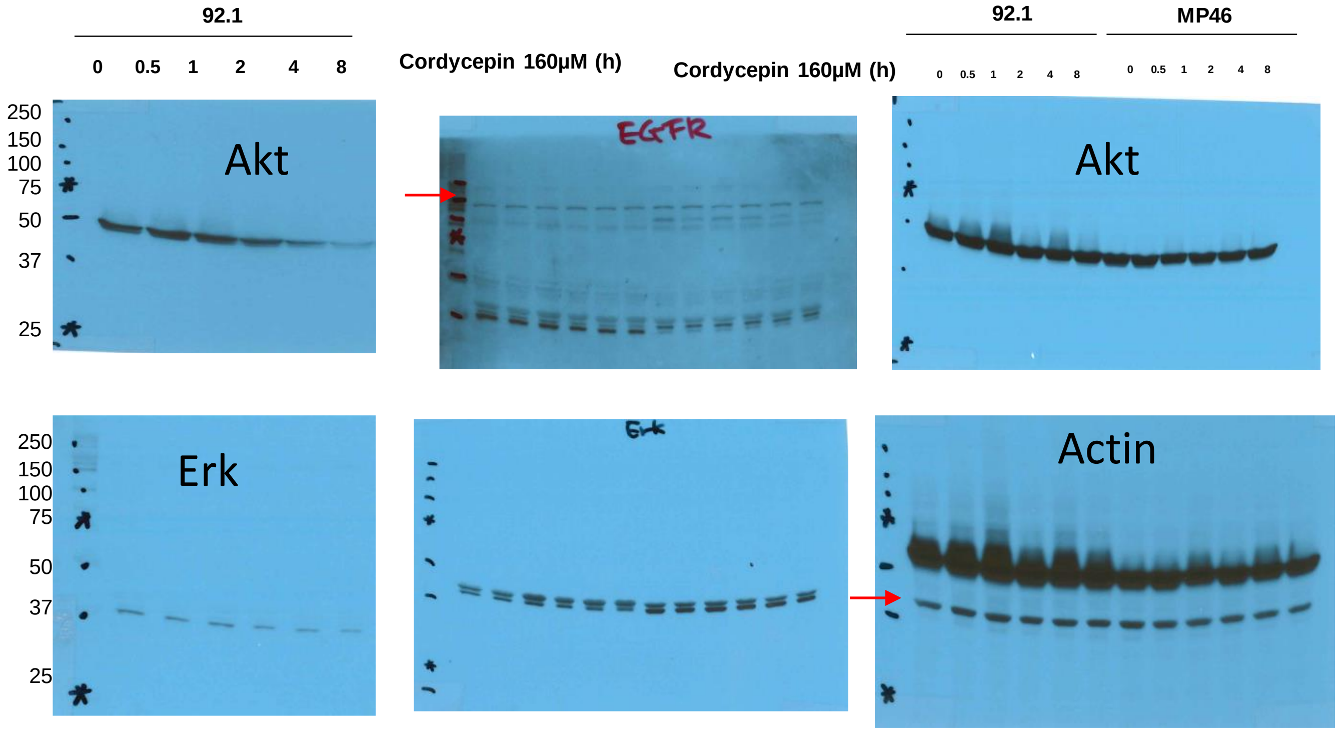

MP46 xeno VEGF

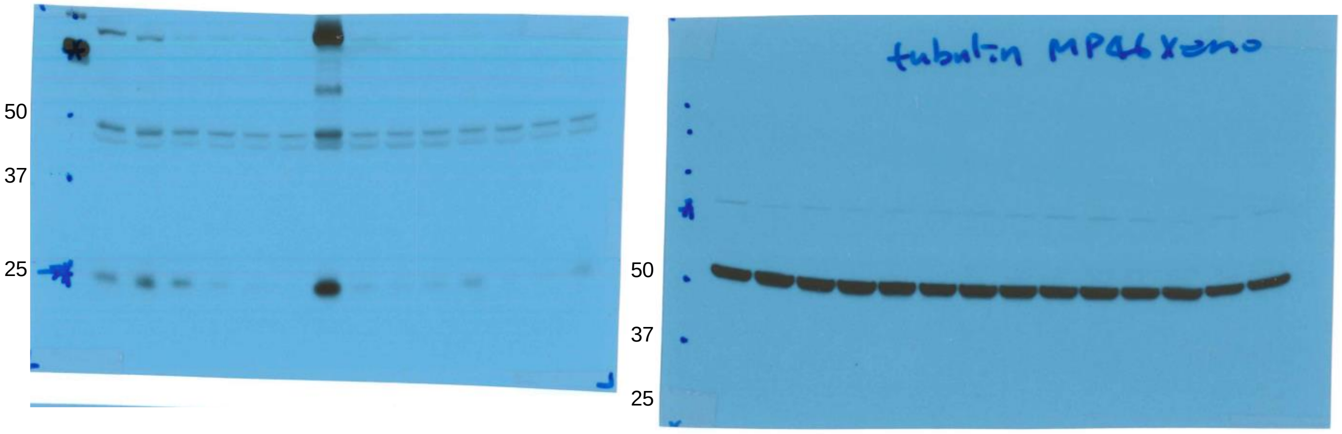

92.1 xeno VEGF

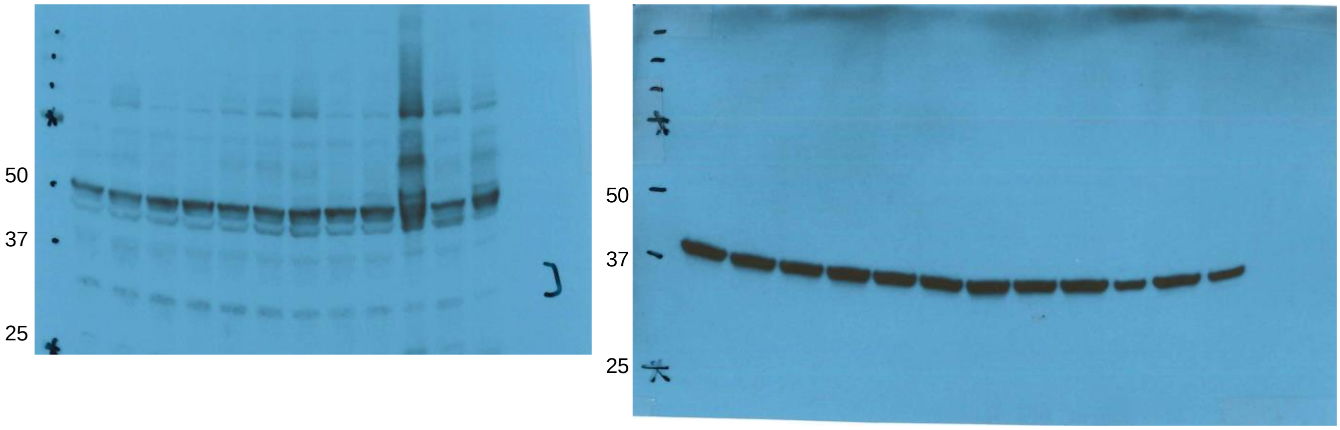

Supplement: Supplementary file 1 [file cancers-14-03122-s001.zip › cancers-1746748-File S1.pdf]
