# Supplementary material for: Cordycepin (3′-Deoxyadenosine) Suppresses Heat Shock Protein 90 Function and Targets Tumor Growth in an Adenosine Deaminase-Dependent Manner
Source: Cancers (Basel). 2022 Jun 25;14(13):3122. doi: 10.3390/cancers14133122 (PMC9264932; doi:10.3390/cancers14133122)
Supplement: Supplementary file 1 [file cancers-14-03122-s001.zip › cancers-1746748-supplementary.pdf]

# Supplemental Figures

## Figure Legends

**Figure S1. Cordycepin inhibits growth of uveal melanoma in an adenosine deaminase (ADA) dependent manner.** **A**, Cell growth assays for cordycepin treatment for 5 days. Uveal melanoma cells are seeded onto 96 cell culture plate and treated with 0, 4, 20 and 40 μM of cordycepin. After 5 days incubation, MTT, or Cell Titer Blue solution was used for measuring fluorescence. **B-C,** Effects of cordycepin treatment on the soft agar colony formation of uveal melanoma cells. **D-E,** the effect of cordycepin on the anchorage-dependent colony formation abilities. **F**, Body weight changes of vehicle- or cordycepin treated mice. 0mg/kg, cordycepin 0mg/kg b.w. treatment; 10mg/kg, cordycepin 10mg/kg b.w. treatment; 20mg/kg, cordycepin 20mg/kg b.w. treatment. Values represent mean ± SD of experiments conducted in sextuplicate. ** $P < 0.01$ and ****$P < 0.0001$ by one-way ANOVA analysis of variance compared with control group.

**Figure S2. Combination treatment with cordycepin and an ADA inhibitor enhances inhibition of anchorage independent-, dependent colony formation and migration abilities in uveal melanoma**
**A**, Cell growth assays for combination treatment with cordycepin and EHNA for 5 days. **B-E,** Effects of combination treatment of cordycepin and EHNA treatment on the anchorage independent- (B) and anchorage dependent colony formation of uveal melanoma cells. **F and G,** Inhibition of migration abilities of uveal melanoma cells by treatment with 10 μM cordycepin and combination treatment of 1μM EHNA (or pentostatin) and 10 μM cordycepin. The cells were seeded onto the Transwells coated with gelatin. After incubation for 12 hours, the migratory cells on the bottom of the membrane were stained with crystal violet solution and counted. Values represent mean ± SD of experiments conducted in sextuplicate. **H,** Body weight changes of vehicle- or compound treated mice. Cordy, cordycepin; CT, control; Pento, pentostatin; Combo1, combination treatment with pentostatin 1mg/kg b.w. and cordycepin 2mg/kg b.w.; Combo2, combination treatment with pentostatin 2mg/kg b.w. and cordycepin 2mg/kg b.w.. Values represent mean ± SEM of experiments conducted in quadruplicate. *** $P < 0.01$ and ****$P < 0.0001$ by one-way ANOVA analysis of variance compared with control group.

**Figure S3. Cordycepin binds Hsp90 and disrupts its function, resulting in degradation of client proteins.**
**A**, 92.1 cells were treated with various combinations of vehicle, 10μM MG-132, and 10 μM cordycepin/1μM of EHNA (combination) then incubated for 6 hours. Expression of ubiquitination was analyzed by Western blot analysis. **B and C**, 92.1 cells were treated with vehicle 10μM MG-132, and 10 μM cordycepin/1μM of EHNA (combination) (**B**), or with various combinations of vehicle, 10μM MG-132, and 160 μM cordycepin (**C**), and then incubated for 6 hours. Total cell lysates were prepared and co- immunoprecipitated (Co-IP) with HiF-1α and anti-mCherry antibodies. The interaction between HiF-1α (or mCherry-Akt1) and Hsp90 was analyzed by Western blot analysis. **D**, Akt1 recombinant protein expressions after treatment of cordycepin in a dose dependent manner in 92.1 and MP46 expressing mCherry-Akt1 recombinant protein. **E**, Cells were treated with 160μM cordycepin for 0, 0.5, 1, 2, 4, 8, 12, and 24 hours. Total Akt, total ERK and EGFR protein expression levels were analyzed by Western blot analysis.

**Figure S4. Antitumor effects of cordycepin in mice xenograft tumor models.**
**A**, VEGF mRNA expression after treatment of 160μM cordycepin. Cells were treated with cordycepin and incubated under 1% $O_2$ conditions for 1.5 hours. **B**, VEGF protein expression in 92.1 and MP46 mice bearded xenograft tumors. CT, control; Combo2, combination treatment with pentostatin 2mg/kg b.w. and cordycepin 2mg/kg b.w.. Values represent mean ± SD of experiments. ****$P < 0.0001$ by one-way ANOVA analysis of variance compared with control group.

## Figure S1.

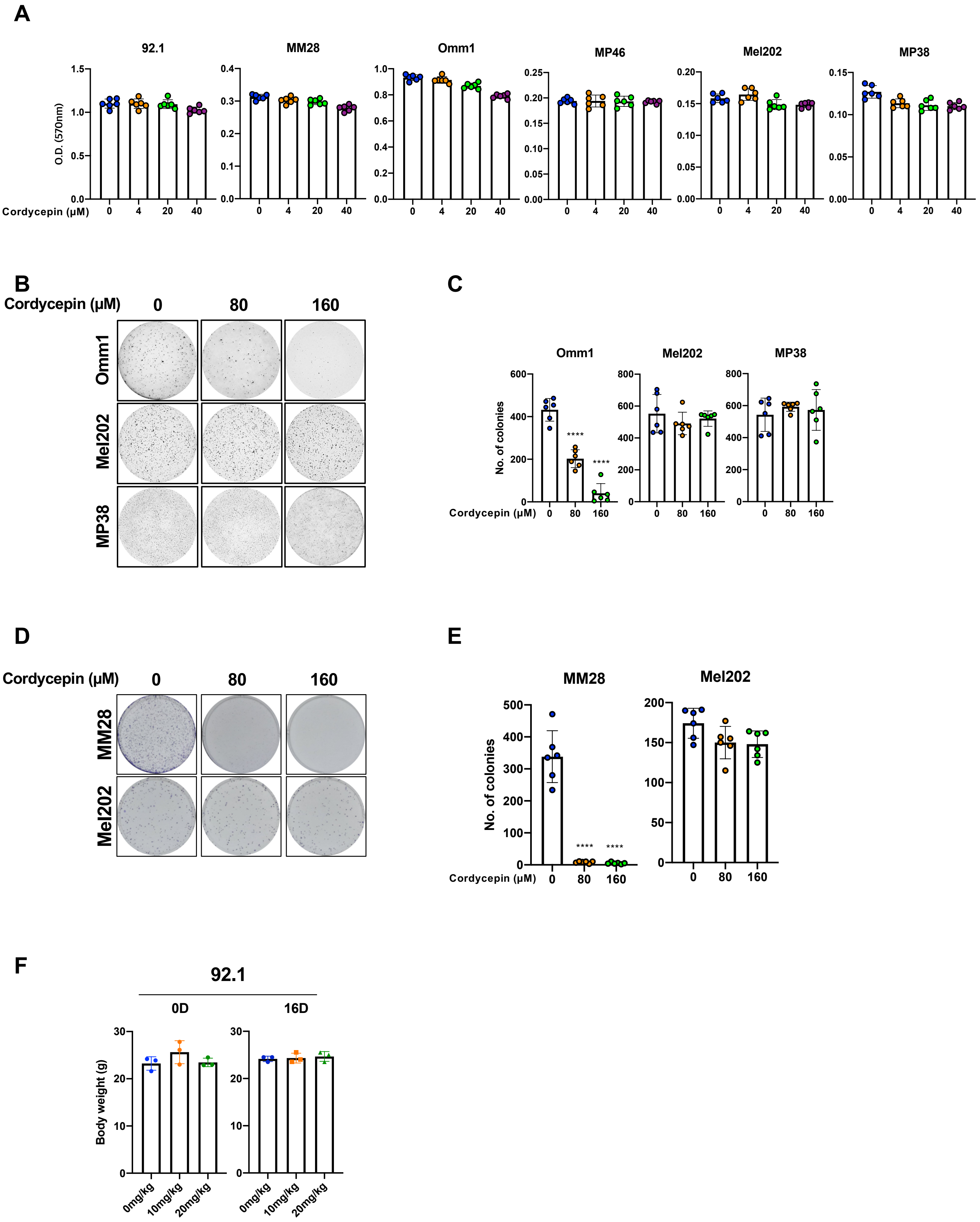
A
92.1
MM28
Omm1
MP46
Mel202
MP38
O.D. (570nm)
Cordycepin (μM)
B
Cordycepin (μM)
0
80
160
Omm1
Mel202
MP38
C
Omm1
Mel202
MP38
No. of colonies
****
Cordycepin (μM)
D
Cordycepin (μM)
MM28
Mel202
E
MM28
Mel202
No. of colonies
Cordycepin (μM)
F
92.1
0D
16D
Body weight (g)
0mg/kg
10mg/kg
20mg/kg

# Figure S2.

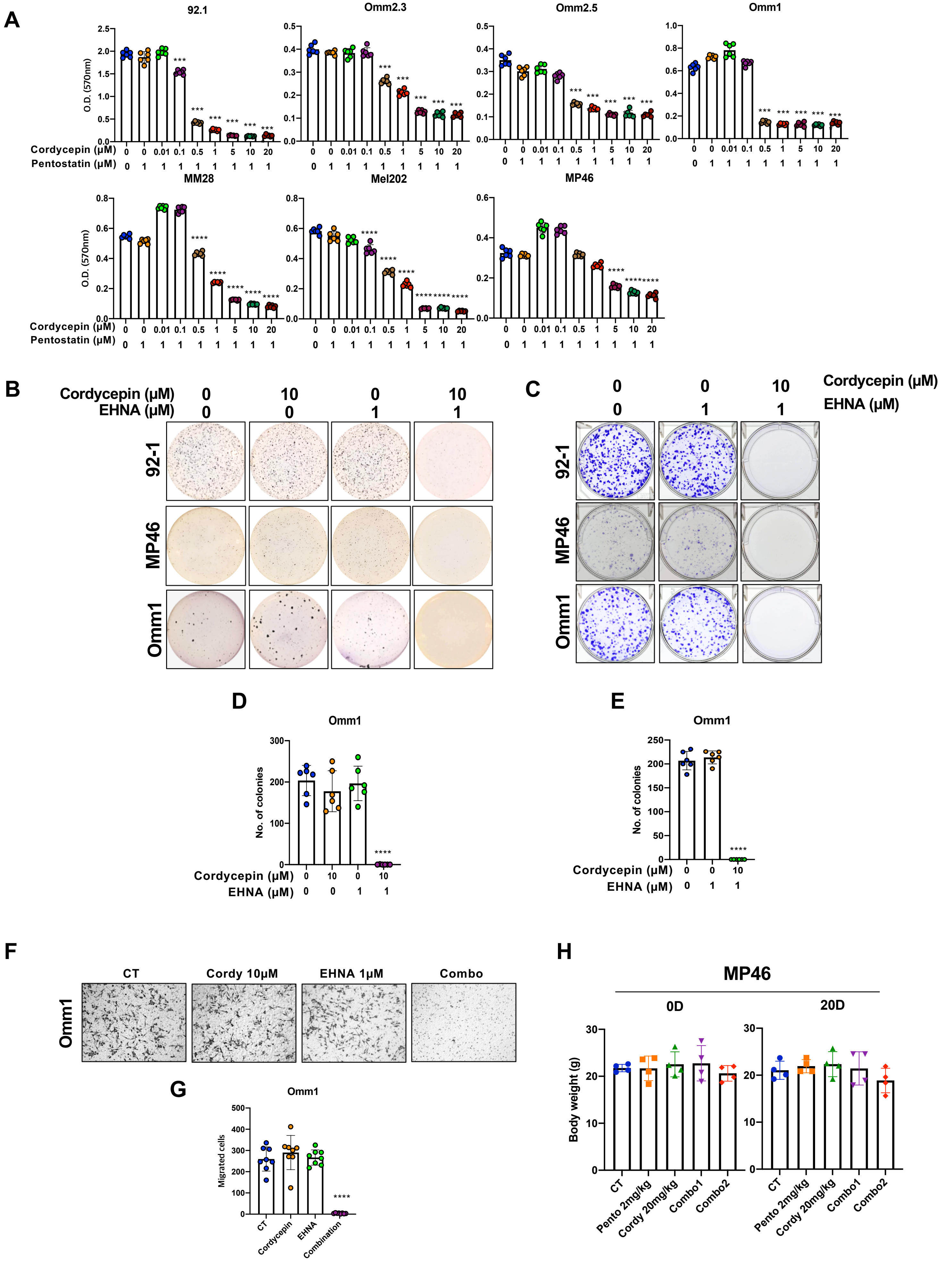
A
92.1
Omm2.3
Omm2.5
Omm1
MM28
Mel202
MP46
O.D. (570nm)
Cordycepin (μM)
Pentostatin (μM)
B
Cordycepin (μM)
EHNA (μM)
92-1
MP46
Omm1
C
D
E
No. of colonies
F
CT
Cordy 10μM
EHNA 1μM
Combo
G
Migrated cells
CT
Cordycepin
EHNA
Combination
H
MP46
0D
20D
Body weight (g)
Pento 2mg/kg
Cordy 20mg/kg
Combo1
Combo2

**Figure S3.**

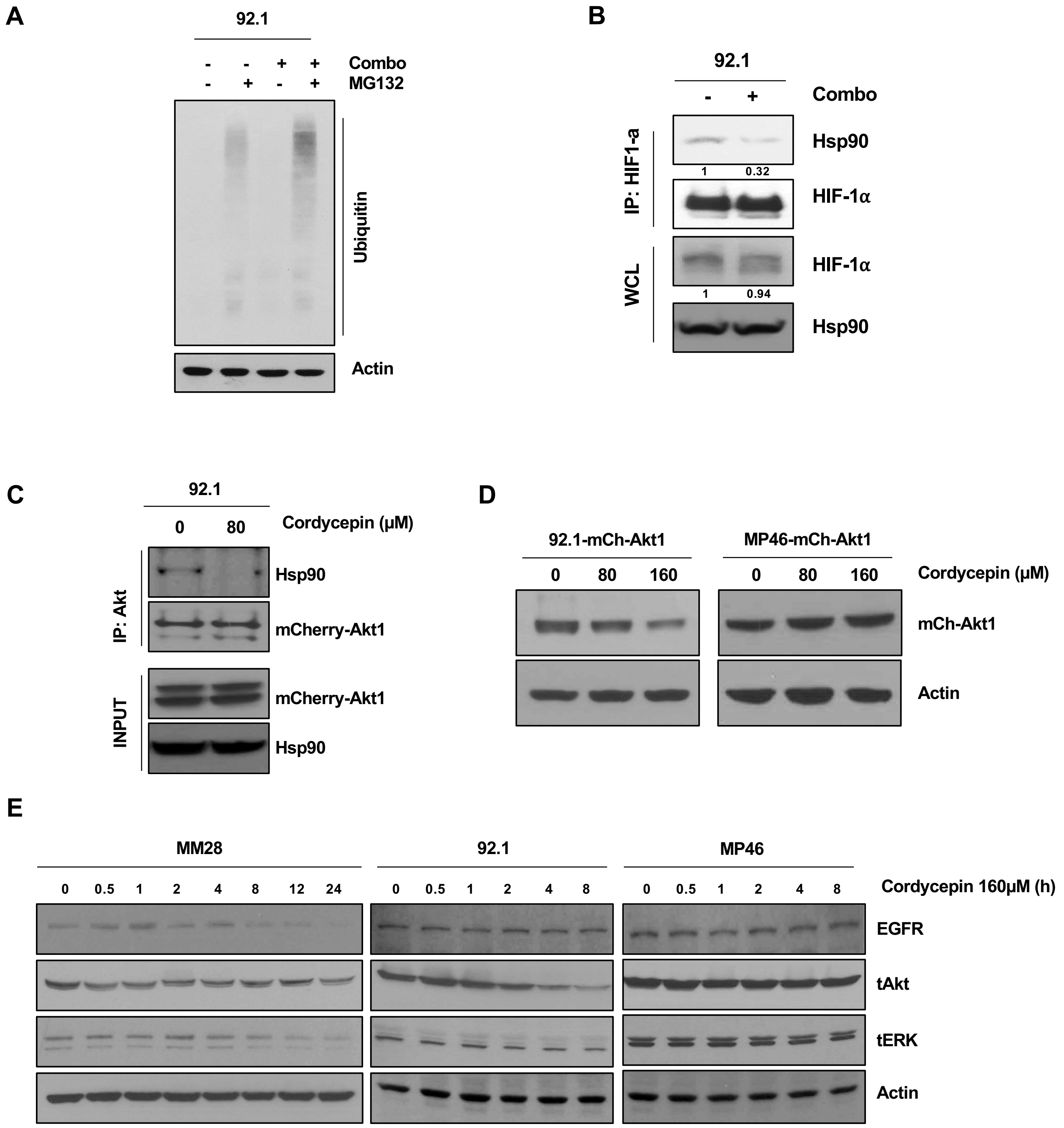

A
92.1
- - + + Combo
- + - + MG132
Ubiquitin
Actin
B
92.1
- + Combo
IP: HIF1-a
Hsp90
1 0.32
HIF-1α
WCL
HIF-1α
1 0.94
Hsp90
C
92.1
0 80 Cordycepin (μM)
IP: Akt
Hsp90
mCherry-Akt1
INPUT
mCherry-Akt1
Hsp90
D
92.1-mCh-Akt1
MP46-mCh-Akt1
0 80 160 0 80 160 Cordycepin (μM)
mCh-Akt1
Actin
E
MM28
92.1
MP46
0 0.5 1 2 4 8 12 24
0 0.5 1 2 4 8
0 0.5 1 2 4 8
Cordycepin 160μM (h)
EGFR
tAkt
tERK
Actin

**Figure S4.**

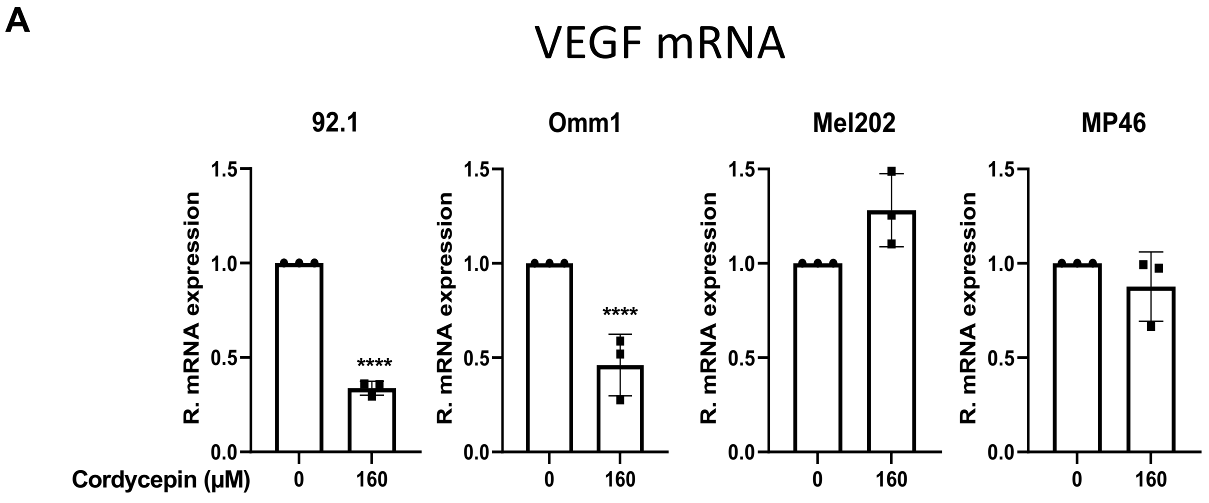
A
VEGF mRNA
92.1
Omm1
Mel202
MP46
R. mRNA expression
1.5
1.0
0.5
0.0
****
****
Cordycepin (μM)
0
160

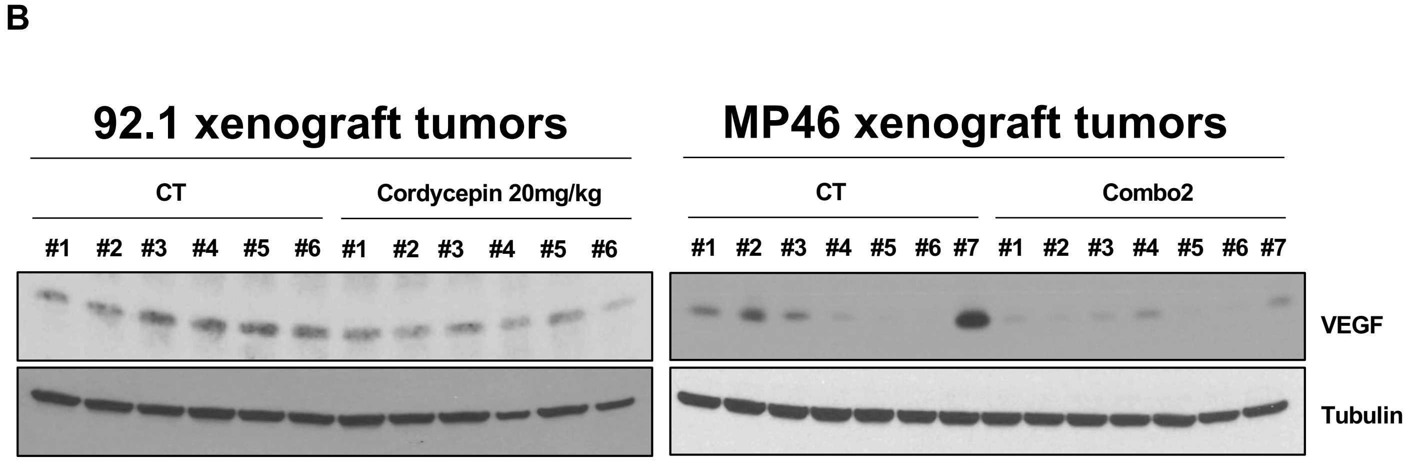
B
92.1 xenograft tumors
MP46 xenograft tumors
CT
Cordycepin 20mg/kg
Combo2
#1 #2 #3 #4 #5 #6 #1 #2 #3 #4 #5 #6
#1 #2 #3 #4 #5 #6 #7 #1 #2 #3 #4 #5 #6 #7
VEGF
Tubulin

**Table S1.**

| 92.1 | Omm1 | MP46 | Mel202 | Omm2.5 | Omm2.3 | PANC1 | MCF7 | BT37 |
|---|---|---|---|---|---|---|---|---|
| 58.46 ± 6.21 | 134.30 ± 15.14 | 258.29 ± 14.71 | 391.12 ± 29.62 | 183.23 ± 7.55 | 190.10 ± 5.63 | 90.91 ± 10.71 | 23.75 ± 1.74 | 36.23 ± 2.34 |

**Table S1. $IC_{50}$ of Cordycepin in various cancer cells**

Determination of $IC_{50}$ of cordycepin in uveal melanoma, pancreatic, breast and AT/RT cancer cell lines. Mean and SD of at least six biological replicates is shown.
